# FastqCleaner: an interactive Bioconductor application for quality-control, filtering and trimming of FASTQ files

**DOI:** 10.1186/s12859-019-2961-8

**Published:** 2019-06-28

**Authors:** Leandro Gabriel Roser, Fernán Agüero, Daniel Oscar Sánchez

**Affiliations:** Instituto de Investigaciones Biotecnológicas (IIB), Universidad Nacional de San Martín - CONICET, 25 de Mayo 1401, B1650HMP San Martín, Buenos Aires Argentina

**Keywords:** Bioconductor, FASTQ, Next generation sequencing, R, Shiny, User-friendly tool, Visualization, Web app

## Abstract

**Background:**

Exploration and processing of FASTQ files are the first steps in state-of-the-art data analysis workflows of Next Generation Sequencing (NGS) platforms. The large amount of data generated by these technologies has put a challenge in terms of rapid analysis and visualization of sequencing information. Recent integration of the R data analysis platform with web visual frameworks has stimulated the development of user-friendly, powerful, and dynamic NGS data analysis applications.

**Results:**

This paper presents *FastqCleaner,* a Bioconductor visual application for both quality-control (QC) and pre-processing of FASTQ files. The interface shows diagnostic information for the input and output data and allows to select a series of filtering and trimming operations in an interactive framework. *FastqCleaner* combines the technology of Bioconductor for NGS data analysis with the data visualization advantages of a web environment.

**Conclusions:**

*FastqCleaner* is an user-friendly, offline-capable tool that enables access to advanced Bioconductor infrastructure. The novel concept of a Bioconductor interactive application that can be used without the need for programming skills, makes *FastqCleaner* a valuable resource for NGS data analysis.

**Electronic supplementary material:**

The online version of this article (10.1186/s12859-019-2961-8) contains supplementary material, which is available to authorized users.

## Background

The advent of Next Generation Sequencing (NGS) technologies has revolutionized genomics, transcriptomics and epigenomics research [1, 2]. The large amount of genetic information produced by these instruments requires suitable data handling and exploration methods. For most common platforms, FASTQ files are the raw starting material for subsequent analyses. A portion of the reads can include adapters or contaminants, the quality of the sequences becomes generally lower towards the end of the reads, and ambiguous base calls may be present. The correction of these and other artifacts are important steps that should be performed before using sequencing reads for mapping or assembly.

Bioconductor [3] is a widely used repository based on the R programming language [4], containing tools devoted to the analysis of high-throughput genomic data. The massive use of these tools is, however, limited by the learning curve that users need to go through to work with customized code routines. Recently, R integration with web tools, in particular JavaScript APIs, has dramatically increased the potential of R to produce more interactive and dynamic experiences of data analysis. This integration is promissory to promote the adoption of R by many researchers for whom learning a programming language has proven to be a prohibitive investment of time and effort.

Here we present *FastqCleaner*, an R package with an offline-capable web application for QC, trimming and filtering of FASTQ files. The tool combines Bioconductor libraries for data analysis and the dynamism of a web application for data visualization.

## Implementation

### Application overview

*FastqCleaner* offers the following features:

1. Implementation of a local, offline-capable and user-friendly web interface.
2. Processing of Single-Read (SR) and Paired-End (PE) files.
3. Dynamic analysis of the input and output files, for customizable sampling size of reads.
4. Interactive, dynamical exploration and visualization of the data, using cutting-edge technology based on JavaScript and CSS3.
5. Cross-platform (running in Linux, Mac-OSX and Windows).
6. Open source, under MIT license.

### Program architecture

*FastqCleaner* was developed in R and is distributed as an R package. Data processing is controlled via R functions, that can be also accessed as normal functions from the R console (Additional file 1). These programs make extensive use of the Bioconductor packages *IRanges* [5], *Biostrings* [6] and *ShortRead* [7]. For speed improvement of the routines, C++ code was implemented in R using the *Rcpp* API [8]. The web interface included in the package was developed with *Shiny* [9], using JavaScript code written via the *jQuery* API, and CSS3.

### Design

**Fig. 1:**
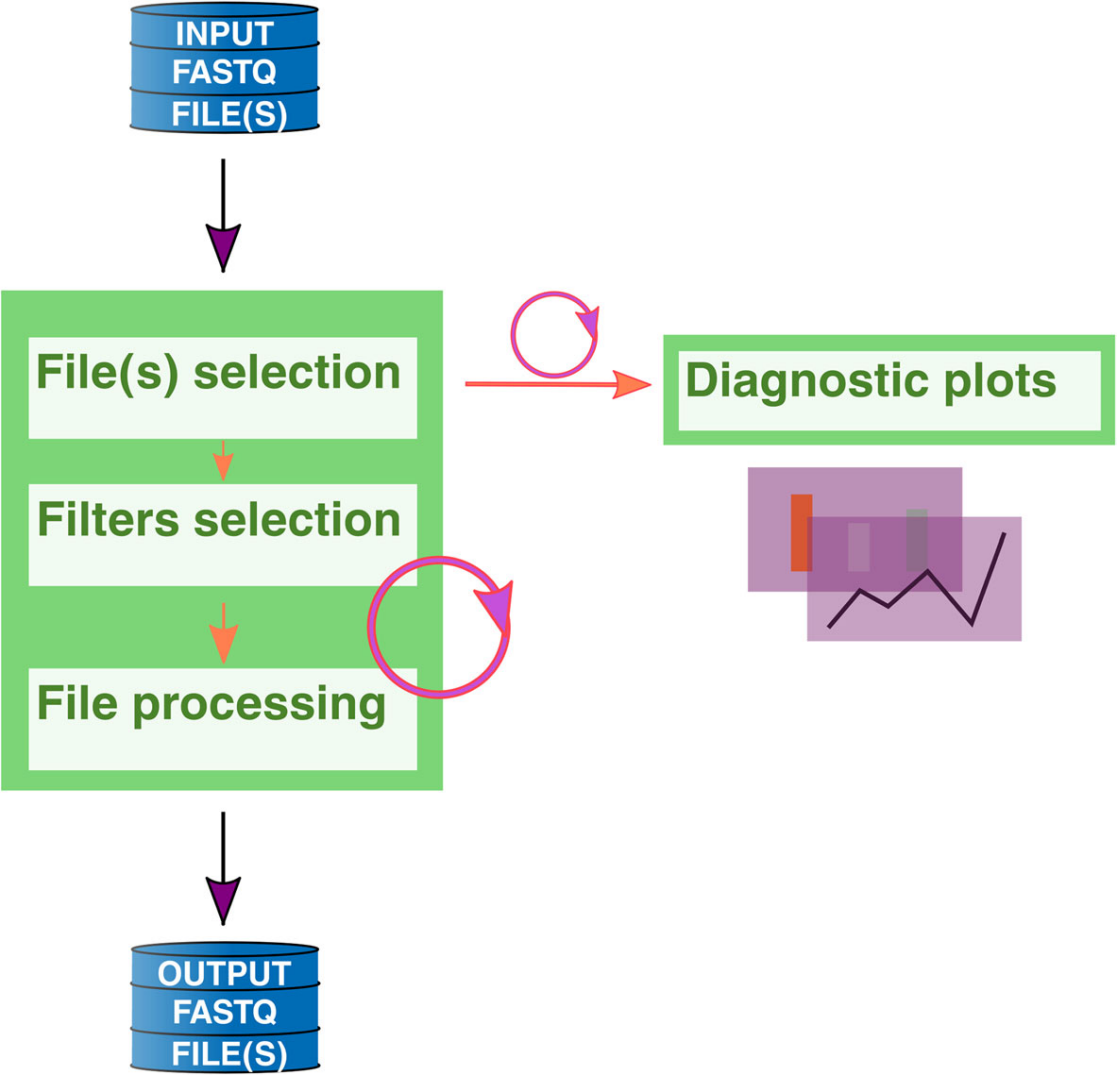
Graphical representation of a typical workflow with *FastqCleaner*, showing the initial selection of FASTQ file(s), processing, and generation of output(s). Diagnostic interactive plots can be constructed for both input and output files. Circular arrows indicate halfway points in the workflow, where different configurations can be selected to re-run the program from there

**Fig. 2:**
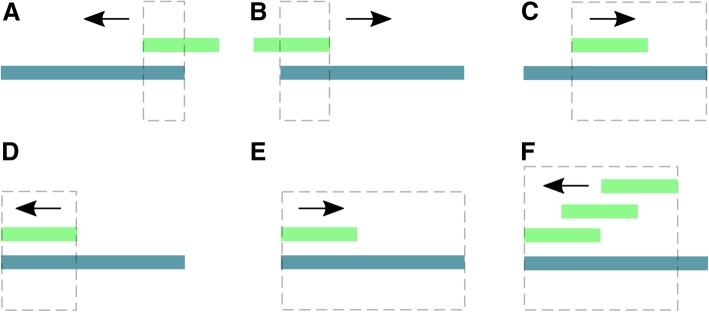
Examples for adapter trimming. Pictures show the relative position of an adapter and a read, and the expected result after processing with the *adapter_filter* function of *FastqCleaner*. Dotted lines indicate the portion of the read that will be removed. Arrows show the direction along the read used for the program to seek for matches. If one or more matches are found, the function trims the longest subsequence, that contains the matching region plus the rest of the read, in the corresponding trimming direction. **a** partial adapter on the right + right-trimming of anchored adapter. **b** partial adapter on the left + left-trimming of anchored adapter. **c** partial adapter within read + right-trimming. *D,E*: full match between an adapter and a portion of the read + left- (**d**) or right- (**e**) trimming. **f** multiple matches for a same adapter + left-trimming

*FastqCleaner* takes compressed or uncompressed SR or PE files as input (Fig. 1). It accepts files with qualities in both Phred+ 33 and Phred+ 64 encoding, detecting Sanger, Solexa and Illumina 1.3+, 1.5+, and > 1.8+ formats. Input files can be processed through a set of independent filters based on either one of the following two principles: 1) *Remotion of a subset of reads that do not meet a given criterion*. This group of filters can remove: a) reads with unknown bases (Ns), b) low complexity sequences, c) duplicated reads, d) reads with length below a threshold quality value, and e) reads with an average quality below a threshold value. 2) *Trimming of individual reads*. This group of filters can trim: a) full and partial adapters, b) 5′ regions below a predefined quality threshold, and c) 3′ or 5′ regions for a fixed nucleotide length. The adapter trimming algorithm extends the methodology of the *trimLRPatterns* function of *Biostrings,* designed to trim on the flanks of reads. For this purpose, *FastqCleaner* includes the *adapter_filter* function, a wrapper of *trimLRPatterns*. The function is able to trim both adapters present on the flanks or within reads (Fig. 2). Several parameters can be passed to modify the behavior of the tool. These parameters allow, for example, to select a different threshold for the number of mismatches, to take into account the presence of indels, etc.

For SR files, *FastqCleaner* sequentially processes a block of reads and writes the resulting post-processed block into the corresponding output file. For PE files, the program uses in each cycle a two-step procedure: first, a block of forward and another of reverse reads are separately processed as in the SR case, and then only those reads present in both post-processed blocks are written into the corresponding output files. Diagnostic plots are constructed using a random sample of reads with customizable size. This feature and the chunk-wise processing methodology mentioned above, allow to work with relatively large files. The current version of *FastqCleaner* supports single-threaded processing. We will add multi-thread support in a future release of the package.

### Availability

The application and a tutorial are available in Bioconductor at https://doi.org/doi:10.18129/B9.bioc.FastqCleaner. Source code for FastqCleaner is also available at GitHub (https://github.com/leandroroser/FastqCleaner) and in Additional file 3.

### Installation

The application can be installed following the instructions detailed at https://doi.org/doi:10.18129/B9.bioc.FastqCleaner

### Launching the application

The application can be launched with the following commands in the R console:

- library("FastqCleaner")
- launch_fqc()

**Fig. 3:**
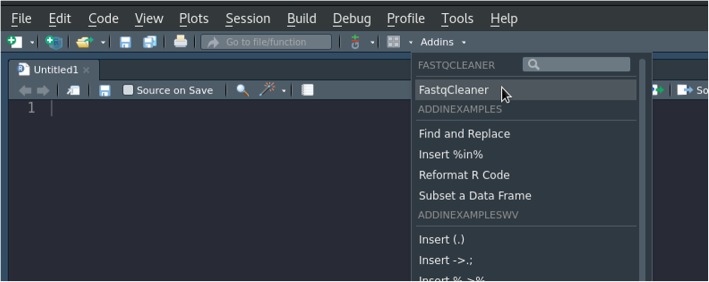
RStudio addins menu, showing the button to launch the *FastqCleaner* application

Optionally, when the application is used in RStudio (versions 0.99.878 or higher), a button that allows the direct launch of the application with a single click can be found in the addins menu (Fig. 3).

## Results

**Fig. 4:**
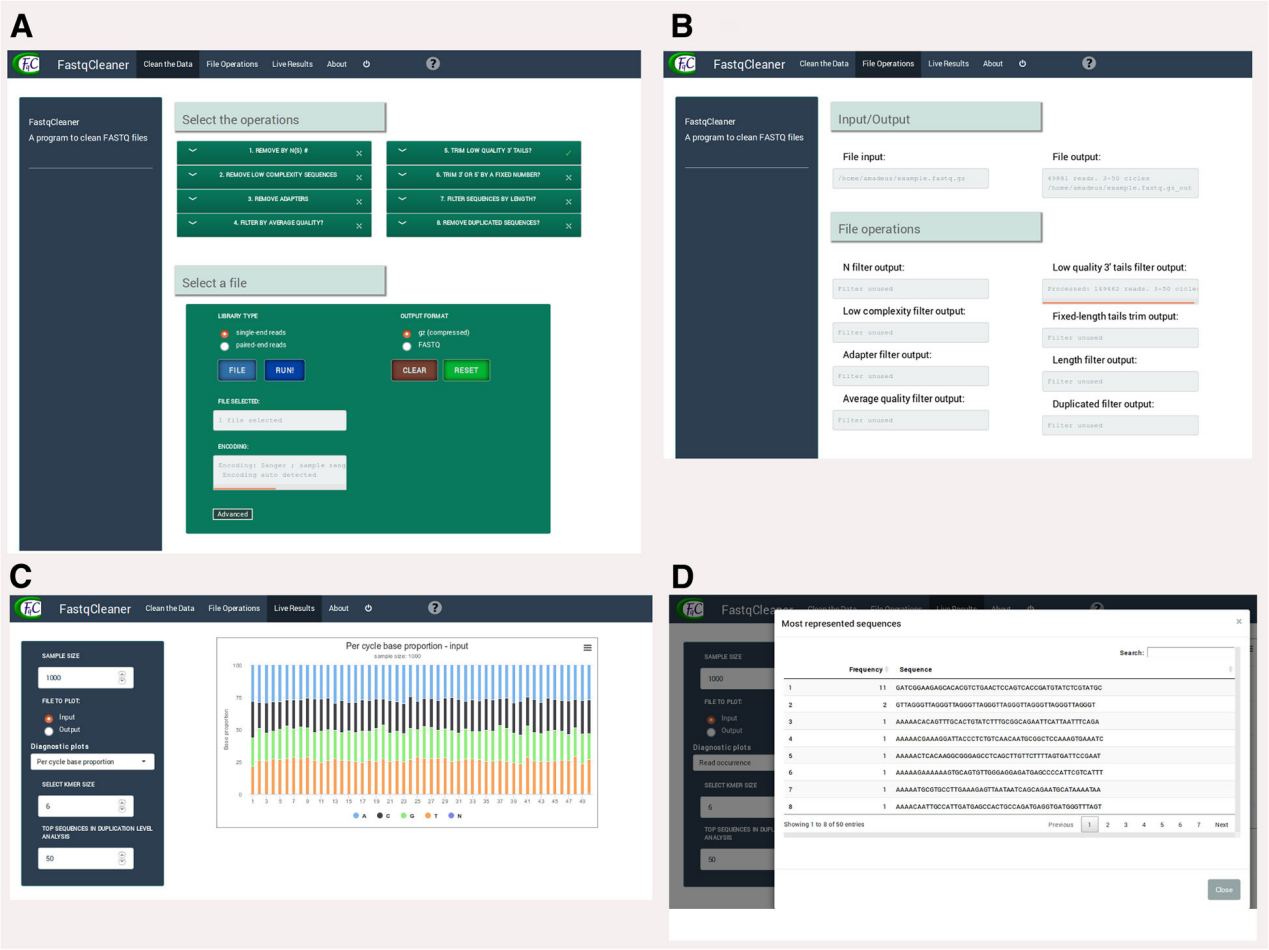
Web interface of the *FastqCleaner* application. **a** first tab, showing an example where a file and a filter are selected. **b** second tab, showing the processes performed after running the program. **c** third tab, showing the analysis of the data, in this case for the input FASTQ file. The plot shows the base composition of the sequences. **d** fourth tab, showing a table with the frequency and the sequence of each duplicated read

The web interface with its three main tabs is described in Fig. 4. The first tab (Fig. 4a) shows the file-selection menu, the available filters, and the run/reset buttons. The selection of files and filters represents the starting point in the *FastqCleaner* workflow. The second tab (Fig. 4b) shows the sequential operations performed on reads after processing. This information consists of the names of the input and output files, and a summary of informative statistics of the reads that passed the filter. The third tab (Figs. 4c, d) shows tables and interactive plots for data diagnostic. Plots can be constructed for both input (original data) and output (post-processed) files. A table with the most frequent k-mers can also be visualized.

**Fig. 5:**
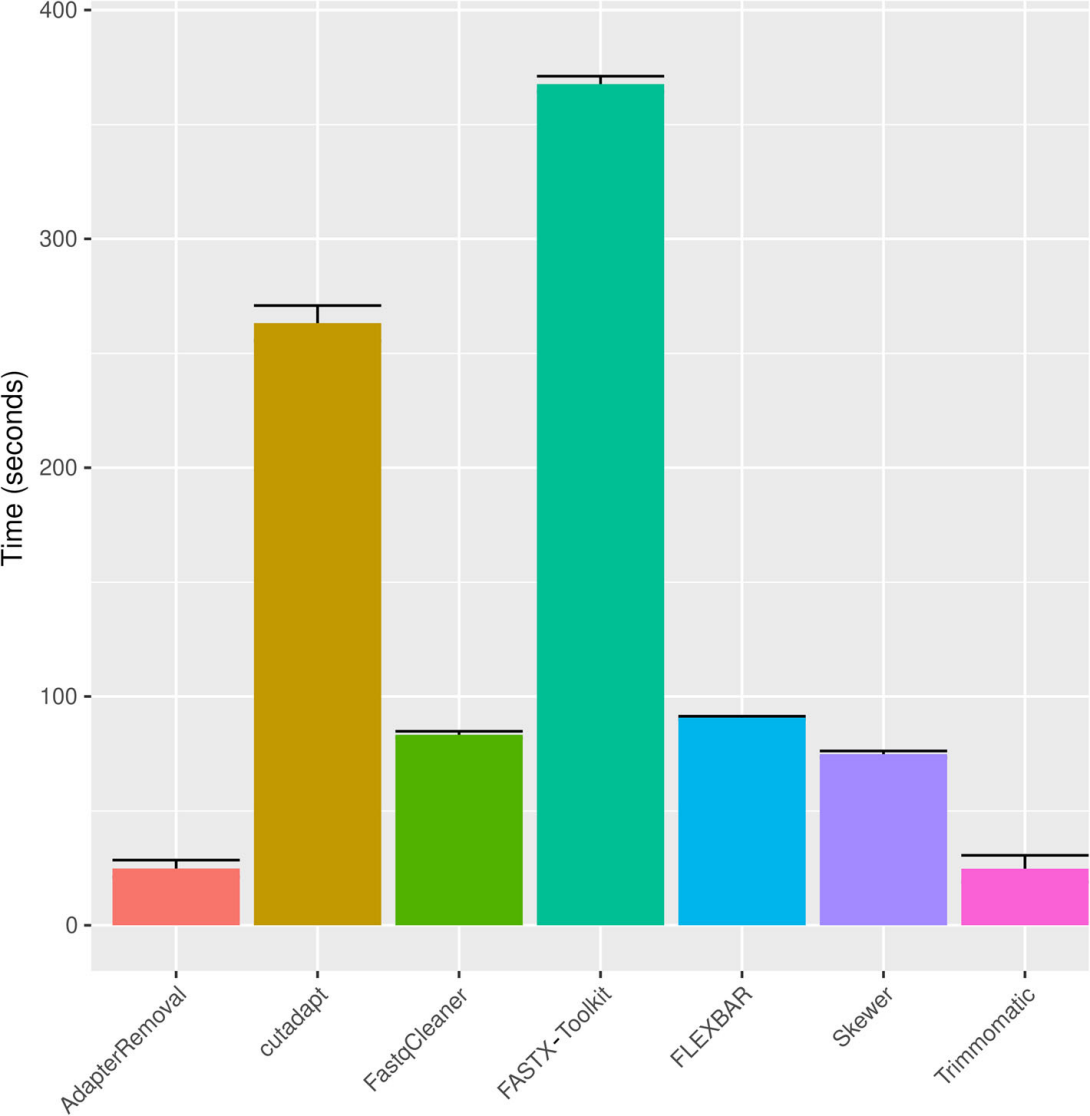
Bar plot for elapsed time (in seconds) for SR adapter trimming and read length filtering

Analysis of SR pre-processing (Fig. 5) showed that the compared tools can be divided into three groups, in function of significant differences observed in processing speed for routine operations (Tukey HSD test, *p* < 0.001 for all the three pairwise comparisons). The slowest were *cutadapt* and *FASTX-Toolkit* (group 1), while *AdapterRemoval* and *Trimmomatic* were the fastest (group 2). *FastqCleaner* showed an intermediate performance, comparable to *Skewer* and *FLEXBAR* (group 3).

**Fig. 6:**
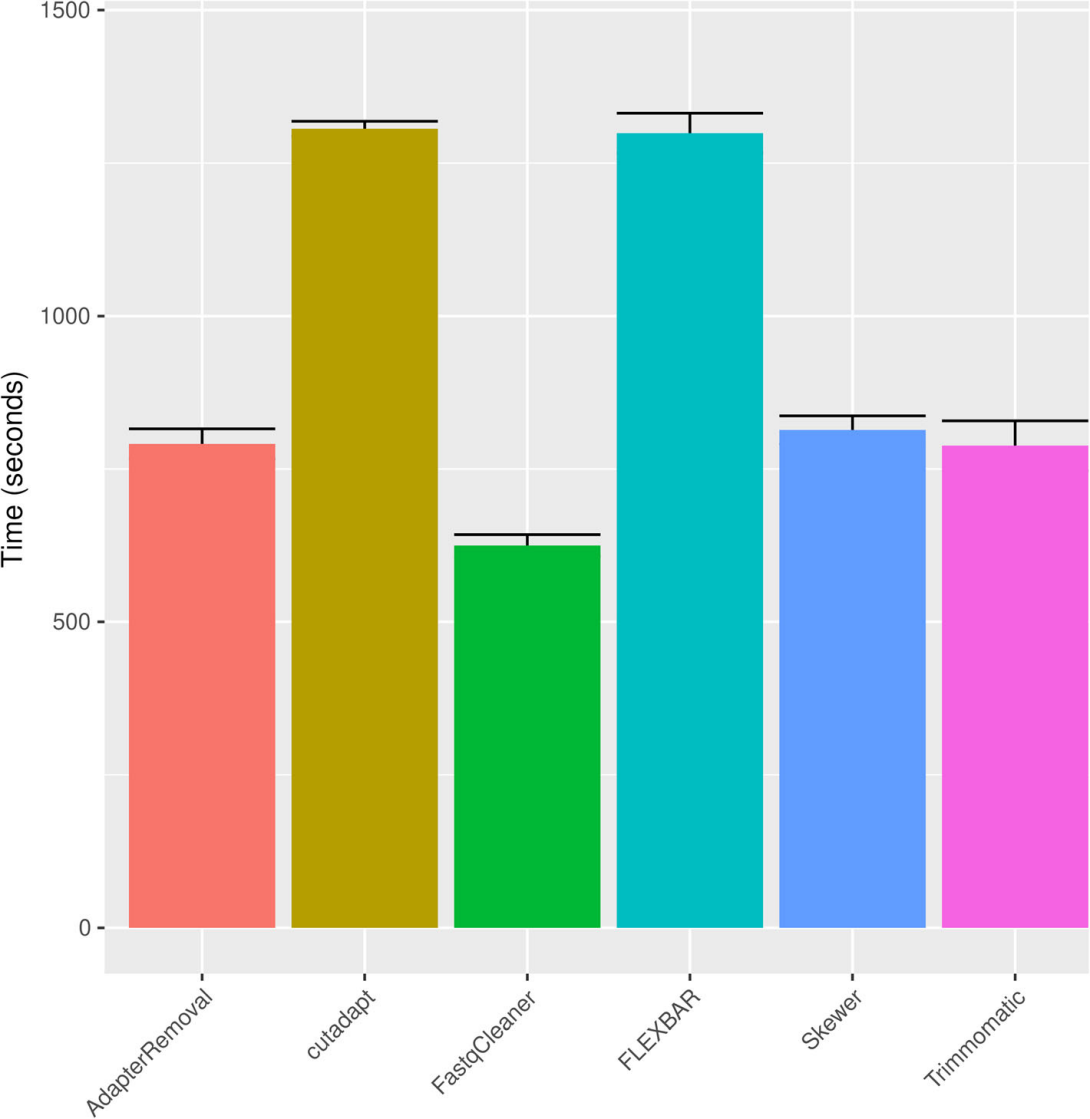
Bar plot for elapsed time (in seconds) for PE adapter trimming and read length filtering. FASTX-Toolkit is not capable to process PE reads and is not shown in the plot

Benchmarking of PE pre-processing operations (Fig. 6) showed that *FastqCleaner* significantly outperforms all other tools for routine operations (Tukey HSD test, p < 0.001 for pairwise comparisons of *FastqCleaner* versus each of the other applications). These results are valid for single-threaded test conditions, and may vary when a multi-thread configuration is used with those programs supporting this feature. Simulated datasets specifically designed for adapter trimming, showed a similar performance in relation to other tools, with a slightly lower performance for some of the computed statistics (Table 1).

**Table 1:** Trimming statistics for simulated reads corresponding to the average results for mate 1 and mate 2

	SEN	SPC	PPV	NVP	MCC
AdapterRemoval	0.999	1.000	1.000	0.999	0.999
Skewer	0.999	1.000	1.000	0.999	0.999
cutadapt	0.907	0.985	0.983	0.914	0.894
FastqCleaner	0.803	0.987	0.985	0.834	0.804

Acronyms: sensitivity (SEN), specificity (SPC), positive predictive value (PPV), negative predictive value (NPV), and Matthews correlation coefficient (MCC)

## Discussion

Benchmarking results for SR pre-processing indicated an excellent performance of *FastqCleaner* in comparison with other tools in terms of elapsed time. Benchmarking of PE pre-processing operations showed that *FastqCleaner* significantly outperforms all other compared tools for routine operations under standardized conditions. Analyses with simulated datasets designed for adapter trimming, also indicated a good overall performance of the application.

## Conclusions

*FastqCleaner* is a tool with a rich and interactive cutting-edge graphical interface for pre-processing and exploration of SR and PE FASTQ files. Comparison with other available programs in a typical pre-processing scenario of adapter trimming and length filtering, showed an excellent performance of the application for both SR and PE real datasets. Simulated datasets designed for trimming operations showed comparable values of the performance statistics, with slightly lower values for some of them. The application is made available as an open source license. Coding experience is not required for its use, and is therefore particularly useful for users who are unfamiliar with R programming. Furthermore, all processing happens locally in the user’s computer (even if the computer is disconnected from the network), making *FastqCleaner* amenable to run in environments where data confidentiality prevents uploading of files to the cloud.

In essence, *FastqCleaner*’s dual capability facilitates both access to the underlying state-of-art Bioconductor infrastructure and to dynamic graphical visualizations in a 100% client-side friendly web environment. This makes *FastqCleaner* a novel technological advance for the analysis of Next Generation Sequencing data.

## Methods

### Experiments on actual data for overall evaluation of the application

In order to assess the performance of *FastqCleaner*, we have compared the package with other available pre-processing tools in benchmark tests: *AdapterRemoval* 2.2.2 [10], *cutadapt* 1.14 [11], *FASTX-Toolkit* 0.0.13 [12], *FLEXBAR* 3.0.3 [13], *Skewer* 0.2.2 [14] and *Trimmomatic* 0.36 [15]. The tests (Additional file 2) were conducted for adapter removal and length filtering using SR and PE files, with 22 replicates of each tests for statistical analysis of performance. Processing conditions were standardized by disabling compression of output files and using a single thread. In addition, pre-processing in *FastqCleaner* was performed using a chunk size of 10,000 reads per cycle. For SR processing, we downloaded from SRA the dataset SRR014966**,** with 14.3 M reads of 36 bp. For PE processing, we downloaded the dataset SRR330569 with 27 M reads of 101 bp. Benchmark tests were conducted in R using a laptop with Linux, a 2.20GHz Intel Core i7 CPU and 16GB of 1600 MHz RAM (Additional file 2).

### Experiments on simulated data for adapter trimming evaluation

We simulated 100 bp PE reads using the following conditions: fold coverage 10X, mean fragment length of 200 bp and standard deviation of 70, *MiSeq2500* reads from the *Escherichia coli* O157:H7 strain (https://www.ncbi.nlm.nih.gov/nuccore/CP014314.1?report=fasta), and right-trimming. The simulations were performed using ART, a NGS read simulator [16]. We used a modified version adapted for simulating adapter-contaminated reads, available at https://sourceforge.net/projects/skewer/files/Simulator/. The trained profile was constructed using the ART-MountRainier version of art_profiler_illumina available at https://www.niehs.nih.gov/research/resources/software/biostatistics/art/index.cfm. This profile was from real *Escherichia coli* O157:H7 isolates (SRA dataset SRR957847). We compared FastqCleaner with other adapter trimming tools. For FastqCleaner, we used error rates between 0.1 and 0.2, averaging the resulting values of the statistics for final data presentation.

Trimming quality was assessed following Lindgreen [17] computing TP (True Positives), the proportion of contaminated reads trimmed to the actual known non-contaminated length, TN (True Negatives), the proportion of untrimmed non-contaminated reads, FP (False Positives), the proportion of over-trimmed reads, and FN (False Negatives), the proportion of under-trimmed reads. Using these four values, the following final statistics were computed: sensitivity [SEN = TP/(TP + FN)], specificity [SPC = TN/(FP + TN)], positive predictive value [PPV = TP/(TP + FP)], negative predictive value [NPV = TN/(TN + FN)], and, as an overall performance measure, the Matthews correlation coefficient {MCC = (TP × TN–FP × FN)/√[(TP + FP) × (TP + FN) × (TN + FP) × (TN + FN)]}. The “generate_data.R” script available as Additional file 4 contains the commands required to generate the simulations used for this part of the paper. The “generate_statistics.R” function available as Additional file 5 defines the code required to compute each of the statistics as per Lindgreen [17].

## Availability and requirements

**Project name:** FastqCleaner.

**Project home page:** https://doi.org/doi:10.18129/B9.bioc.FastqCleaner

**Operating system(s):** Platform independent.

**Programming language:** R, C++, HTML, JavaScript, CSS3.

**Other requirements:** R > = 3.6.0.

**License:** MIT.

**Any restrictions to use by non-academics**: Not applicable.

## Additional files

Additional file 1: PDF version of the online tutorial. (PDF 1048 kb)

Additional file 2: R script used in this work for benchmark testing. (R 3 kb)

Additional file 3: Source code of *FastqCleaner*. (GZ 3273 kb)

Additional file 4: R script to compute simulated data using ART for *Escherichia coli* O157:H7 data. (R 3 kb)

Additional file 5: R script to compute statistics for the different trimming tools as per *Lindgreen* (2017). (R 970 bytes)

## Data Availability

*FastqCleaner* is freely available from its Bioconductor home page at https://doi.org/doi:10.18129/B9.bioc.FastqCleaner under MIT license. *FastqCleaner* can be launched on any system that has R installed. An online tutorial is available at the package home page. A PDF version of this tutorial is included as supplemental material (Additional file 1). Source code for FastqCleaner is also available at GitHub (https://github.com/leandroroser/FastqCleaner) and in Additional file 3.

## Abbreviations

NGS: Next Generation Sequencing
PE: Paired-End Reads
SR: Single-Reads
