# Supplementary material for: FastqCleaner: an interactive Bioconductor application for quality-control, filtering and trimming of FASTQ files
Source: BMC Bioinformatics. 2019 Jun 28;20:361. doi: 10.1186/s12859-019-2961-8 (PMC6599294; doi:10.1186/s12859-019-2961-8)
Supplement: Supplementary file 3 — Source code of FastqCleaner. (GZ 3273 kb) [file 12859_2019_2961_MOESM3_ESM.gz › FastqCleaner/inst/application/www/help/docs/authors.html]

Authors • FastqCleaner


FastqCleaner
0.99.28

- Reference

# Authors

- **Leandro Roser**. Author, maintainer.
- **Fernán Agüero**. Author.
- **Daniel Sánchez**. Author.

Developed by Leandro Roser, Fernán Agüero, Daniel Sánchez.

Site built with pkgdown.
