# Supplementary material for: FastqCleaner: an interactive Bioconductor application for quality-control, filtering and trimming of FASTQ files
Source: BMC Bioinformatics. 2019 Jun 28;20:361. doi: 10.1186/s12859-019-2961-8 (PMC6599294; doi:10.1186/s12859-019-2961-8)
Supplement: Supplementary file 3 — Source code of FastqCleaner. (GZ 3273 kb) [file 12859_2019_2961_MOESM3_ESM.gz › FastqCleaner/inst/application/www/help/docs/index.html]

A Shiny Application for Quality Control, Filtering and Trimming of FASTQ Files • FastqCleaner
 

 


 


FastqCleaner
0.99.28

- Reference

# FastqCleaner

A Shiny web-app to clean FASTQ files with R and Bioconductor

## 1. Prerequisites and dependences for the direct installation of this GitHub repository

#### Linux users

- Libraries OpenSSL and libcurl installed

-> Ubuntu:

Paste the following command in terminal:

```
sudo apt-get install libssl-dev libcurl4-openssl-dev
```

#### Mac users

- Command Line Tools

Type in a Terminal:

```
xcode-select --install
```

A software update popup will ask if the Command Line Tools must be installed

- XQuartz

XQuartz can be downloaded from the following link: https://www.xquartz.org/

#### Windows users

- Rtools

The program is available at https://cran.r-project.org/bin/windows/Rtools/

## 2. Installation

Paste the following command in the R console:

```
source("https://goo.gl/u2xraS")
```

## 3. Launching the application

```
library("FastqCleaner")
launch_fqc()
```

## 4. Description and usage

See the tutorial of the application in  this link

## Links

- Report a bug at   
  learoser@gmail.com

## License

- GPL (>= 2)

## Developers

- Leandro Roser   
   Author, maintainer
- Fernán Agüero   
   Author
- Daniel Sánchez   
   Author

Developed by Leandro Roser, Fernán Agüero, Daniel Sánchez.

Site built with pkgdown.
