# Supplementary material for: FastqCleaner: an interactive Bioconductor application for quality-control, filtering and trimming of FASTQ files
Source: BMC Bioinformatics. 2019 Jun 28;20:361. doi: 10.1186/s12859-019-2961-8 (PMC6599294; doi:10.1186/s12859-019-2961-8)
Supplement: Supplementary file 3 — Source code of FastqCleaner. (GZ 3273 kb) [file 12859_2019_2961_MOESM3_ESM.gz › FastqCleaner/inst/application/www/help/docs/reference/adapter_filter.html]

Remove full and partial adapters from a ShortReadQ object — adapter\_filter • FastqCleaner


FastqCleaner
0.99.28

- Reference
- Articles
  - An Introduction to FastqCleaner

# Remove full and partial adapters from a ShortReadQ object

`adapter_filter.Rd`

This program can remove adapters and partial
adapters from 3' and 5', using the functions
`isMatchingEndingAt` and
`isMatchingStartingAt`
of Biostrings. The program extends the methodology of
the `trimLRPatterns` function of Biostrings,
being also capable of removing adapters present within reads.
For a given position in the read, the two Biostrings functions return TRUE
when a match is present between a substring of the read and the adapter.
As `trimLRPatterns`, adapter\_filter also selects
region and goes up to the end of the sequence in the corresponding flank
as the best match.
If several valid matches are found, the function removes the
largest subsequence. Adapters can be anchored or not. When indels are allowed,
the second method uses the 'edit distance' between the subsequences
and the adapter

```
adapter_filter(input, Lpattern = "", Rpattern = "", method = c("exact",
  "er"), rc.L = FALSE, rc.R = FALSE, first = c("R", "L"),
  with_indels = FALSE, error_rate = 0.2, anchored = TRUE,
  fixed = "subject", remove_zero = TRUE, checks = TRUE,
  min_match_flank = 1L, ...)
```

## Arguments

| input | `ShortReadQ` object |
| Lpattern | 5' pattern (character or `DNAString` object) |
| Rpattern | 3' pattern (character or `DNAString` object) |
| method | Method used for trimming. If 'exact' the method is based on the exact match between the possible subsequences of the subject and adapter(s). If 'er' the metod is based on the error-rate between the subsequences, allowing mismatches in any place |
| rc.L | Reverse complement Lpattern? default FALSE |
| rc.R | Reverse complement Rpatter? default FALSE |
| first | trim first right('R') or left ('L') side of sequences when both Lpattern and Rpattern are passed |
| with\_indels | Allow indels? This feature is available only when the error\_rate is not null |
| error\_rate | Error rate (value in the range [0, 1] used for the 'er' method. The error rate is the proportion of mismatches allowed between the adapter and the aligned portion of the subject. For a given adapter A, the number of allowed mismatches between each subsequence s of A and the subject is computed as: error\_rate \* L\_s, where L\_s is the length of the subsequence s |
| anchored | Adapter or partial adapter within sequence (anchored = FALSE, default) or only in 3' and 5' terminals? (anchored = TRUE) |
| fixed | Parameter passed to `isMatchingStartingAt` or `isMatchingEndingAt`. Default 'subject', where only ambiguities in the pattern are interpreted as wildcard |
| remove\_zero | Remove zero-length sequences? Default TRUE |
| checks | Perform checks? Default TRUE |
| min\_match\_flank | When a match is found between a subsequence of the subject and the adapter in the corresponding flank, which would be the minimum length of the overlapping region (threshold) used for trimming? Default is 1L (trim when >= 2 base(s) match). |
| ... | additional parameters passed to `isMatchingStartingAt` or `isMatchingEndingAt`. |

## Value

Filtered `ShortReadQ`
object

## Examples

```
require('Biostrings')
require('ShortRead')

# create 6 sequences of width 43
set.seed(10)
input <- random_seq(6, 43)

# add adapter in 3' reverse complemented. In read 1, 
# it will appear the 5' adapter of read 2 reverse complemented.
adapter <- 'ATCGACT'

input <- paste0(input, as.character(reverseComplement(DNAString(adapter))))
input <- DNAStringSet(input)

# create qualities of width 50
set.seed(10)
input_q <- random_qual(c(30,40), slength = 6, swidth = 50,
encod = 'Sanger')

# create names
input_names <- seq_names(length(input))

# create ShortReadQ object
my_read <- ShortReadQ(sread = input, quality = input_q, id = input_names)

# trim adapter
filtered <- adapter_filter(my_read, Rpattern = adapter, rc.R = TRUE)

# look at the filtered sequences
sread(filtered)


#>   A DNAStringSet instance of length 6
#>     width seq
#> [1]    43 TGGTCCGGTGTTCTGGCGGAATAGGTACAGTCCAGTAATTGCC
#> [2]    43 TCCCGCAGACGCTGGGTCCGGAATGCCCTTTCTGAGCAGCTCC
#> [3]    43 AGCCGTTTGACTTCGCGGAAAGTGAACTTAGATTCGGTCCTGA
#> [4]    43 AACACGGTACTTCCACAGTCAACCCGCCGACTTGGAGAATTTA
#> [5]    43 TTAGCCGGGCGGTTATTCCCCTAGTGATCTTACTAAGATTTGC
#> [6]    43 AATACCTAAGCGAAGTGACAGATATGTTCGTCATTCATCCAGG


# adapter in the second strand of paired-end reads is reverse-complemented,
# with adapter in the end of sequence
adapterR <- as.character(reverseComplement(DNAString('ATCGACT')))
adapterR <- DNAString(adapterR)
inputR <- reverseComplement(input)


# create qualities of width 50
set.seed(10)
inputqR <- random_qual(c(30,40), slength = 6, swidth = 50,
encod = 'Sanger')

my_readR <- ShortReadQ(sread = inputR, quality = inputqR, id = input_names)

# trim adapter
filteredR <- adapter_filter(my_readR, Rpattern = adapterR)

# look at the filtered sequences
sread(filteredR)


#>   A DNAStringSet instance of length 6
#>     width seq
#> [1]    50 ATCGACTGGCAATTACTGGACTGTACCTATTCCGCCAGAACACCGGACCA
#> [2]    50 ATCGACTGGAGCTGCTCAGAAAGGGCATTCCGGACCCAGCGTCTGCGGGA
#> [3]    50 ATCGACTTCAGGACCGAATCTAAGTTCACTTTCCGCGAAGTCAAACGGCT
#> [4]    50 ATCGACTTAAATTCTCCAAGTCGGCGGGTTGACTGTGGAAGTACCGTGTT
#> [5]    50 ATCGACTGCAAATCTTAGTAAGATCACTAGGGGAATAACCGCCCGGCTAA
#> [6]    50 ATCGACTCCTGGATGAATGACGAACATATCTGTCACTTCGCTTAGGTATT
```

## Contents

- Arguments
- Value
- Examples

## Author

Leandro Roser learoser@gmail.com

Developed by Leandro Roser, Fernán Agüero, Daniel Sánchez.

Site built with pkgdown.
