# Supplementary material for: FastqCleaner: an interactive Bioconductor application for quality-control, filtering and trimming of FASTQ files
Source: BMC Bioinformatics. 2019 Jun 28;20:361. doi: 10.1186/s12859-019-2961-8 (PMC6599294; doi:10.1186/s12859-019-2961-8)
Supplement: Supplementary file 3 — Source code of FastqCleaner. (GZ 3273 kb) [file 12859_2019_2961_MOESM3_ESM.gz › FastqCleaner/inst/application/www/help/docs/reference/check_encoding.html]

Check quality encoding — check\_encoding • FastqCleaner


FastqCleaner
0.99.28

- Reference
- Articles
  - An Introduction to FastqCleaner

# Check quality encoding

`check_encoding.Rd`

Check quality encoding

```
check_encoding(x = NULL, custom = NULL)
```

## Arguments

| x | Quality values |
| custom | custom encoding from the following:  'Sanger' --------> expected range: [0, 40]  'Illumina1.8' --------> expected range: [0, 41]  'Illumina1.5' --------> expected range: [0, 40]  'Illumina1.3' --------> expected range: [3, 40]  'Solexa' --------> expected range: [-5, 40] |

## Value

List with encoding information

## Examples

```
require(Biostrings)

x <- list(PhredQuality(0:40), SolexaQuality(-5:40), IlluminaQuality(3:40))
x <- lapply(x, function(i)utf8ToInt(as.character(i)[1]))
lapply(x, check_encoding)


#> [[1]]
#> [[1]]$x
#> [1] "Sanger"
#> 
#> [[1]]$y
#> [1] 1
#> 
#> [[1]]$q
#> [1] 33
#> 
#> [[1]]$range
#> [1] "[33 - 73]"
#> 
#> 
#> [[2]]
#> [[2]]$x
#> [1] "Solexa"
#> 
#> [[2]]$y
#> [1] 5
#> 
#> [[2]]$q
#> [1] 64
#> 
#> [[2]]$range
#> [1] "[59; 104]"
#> 
#> 
#> [[3]]
#> [[3]]$x
#> [1] "Illumina 1.5+"
#> 
#> [[3]]$y
#> [1] 3
#> 
#> [[3]]$q
#> [1] 64
#> 
#> [[3]]$range
#> [1] "[66; 104]"
#> 
#>


SolexaQuality(0:40)


#>   A SolexaQuality instance of length 1
#>     width seq
#> [1]    41 @ABCDEFGHIJKLMNOPQRSTUVWXYZ[\]^_`abcdefgh


IlluminaQuality(0:40)


#>   A IlluminaQuality instance of length 1
#>     width seq
#> [1]    41 @ABCDEFGHIJKLMNOPQRSTUVWXYZ[\]^_`abcdefgh
```

## Contents

- Arguments
- Value
- Examples

## Author

Leandro Roser learoser@gmail.com

Developed by Leandro Roser, Fernán Agüero, Daniel Sánchez.

Site built with pkgdown.
