# Supplementary material for: FastqCleaner: an interactive Bioconductor application for quality-control, filtering and trimming of FASTQ files
Source: BMC Bioinformatics. 2019 Jun 28;20:361. doi: 10.1186/s12859-019-2961-8 (PMC6599294; doi:10.1186/s12859-019-2961-8)
Supplement: Supplementary file 3 — Source code of FastqCleaner. (GZ 3273 kb) [file 12859_2019_2961_MOESM3_ESM.gz › FastqCleaner/inst/application/www/help/docs/reference/complex_filter.html]

Remove sequences with low complexity — complex\_filter • FastqCleaner


FastqCleaner
0.99.28

- Reference
- Articles
  - An Introduction to FastqCleaner

# Remove sequences with low complexity

`complex_filter.Rd`

The program removes low complexity sequences, computing the
entropy with the observed frequency of dinucleotides.

```
complex_filter(input, threshold = 0.5, referenceEntropy = 3.908135)
```

## Arguments

| input | `ShortReadQ` object |
| threshold | A threshold value computed as the relation of the H of the sequences and the reference H. Default is 0.5 |
| referenceEntropy | Reference entropy. By default, the program uses a value of 3.908, that corresponds to the entropy of the human genome in bits |

## Value

Filtered `ShortReadQ`
object

## Examples

```
require('Biostrings')
require('ShortRead')

# create  sequences of different width
set.seed(10)
input <- lapply(c(0, 6, 10, 16, 20, 26, 30, 36, 40),
               function(x) random_seq(1, x))


# create repetitive 'CG' sequences with length adequante 
# for a total length:
# input +  CG = 40

CG <- lapply(c(20, 17, 15, 12, 10, 7, 5, 2, 0),
            function(x) paste(rep('CG', x), collapse = ''))


# concatenate input and CG
input  <- mapply('paste', input, CG, sep = '')
input <- DNAStringSet(input)

# plot relative entropy (E, Shannon 1948)

freq <- dinucleotideFrequency(input)
freq  <- freq /rowSums(freq)
H <- -rowSums(freq  * log2(freq), na.rm = TRUE)
H_max <- 3.908135  # max entropy
plot(H/H_max, type='b', xlab = 'Sequence', ylab= 'E')


# create qualities of width 40

input_q <- random_qual(c(30,40), slength = 9, swidth = 40,
                       seed = 10, encod = 'Sanger')


#> Error in random_qual(c(30, 40), slength = 9, swidth = 40, seed = 10, encod = "Sanger"): unused argument (seed = 10)


# create names
input_names <- seq_names(9)

# create ShortReadQ object
my_read <- ShortReadQ(sread = input, quality = input_q, id = input_names)


#> Error in ShortReadQ(sread = input, quality = input_q, id = input_names): objeto 'input_q' no encontrado


# apply the filter
filtered <- complex_filter(my_read)


#> Error in sread(input): objeto 'my_read' no encontrado


# look at the filtered sequences
sread(filtered)


#> Error in sread(filtered): objeto 'filtered' no encontrado
```

## Contents

- Arguments
- Value
- Examples

## Author

Leandro Roser learoser@gmail.com

Developed by Leandro Roser, Fernán Agüero, Daniel Sánchez.

Site built with pkgdown.
