# Supplementary material for: FastqCleaner: an interactive Bioconductor application for quality-control, filtering and trimming of FASTQ files
Source: BMC Bioinformatics. 2019 Jun 28;20:361. doi: 10.1186/s12859-019-2961-8 (PMC6599294; doi:10.1186/s12859-019-2961-8)
Supplement: Supplementary file 3 — Source code of FastqCleaner. (GZ 3273 kb) [file 12859_2019_2961_MOESM3_ESM.gz › FastqCleaner/inst/application/www/help/docs/reference/create_uniform_width.html]

Create fastq/sequences/qualities with uniform width — create\_uniform\_width • FastqCleaner


FastqCleaner
0.99.28

- Reference
- Articles
  - An Introduction to FastqCleaner

# Create fastq/sequences/qualities with uniform width

`create_uniform_width.Rd`

Create fastq/sequences/qualities with uniform width

```
create_uniform_width(input, type = c("fastq", "sequence", "quality"))
```

## Arguments

| input | input to edit |
| type | type of the input: 'fastq' (ShortReadQ), 'sequence' (DNAStringSet), 'quality' (BStringset) |

## Value

ShortReadQ object or character vector with sequences
or qualities, with uniform widht (padded with Ns or })

## Contents

- Arguments
- Value

Developed by Leandro Roser, Fernán Agüero, Daniel Sánchez.

Site built with pkgdown.
