# Supplementary material for: FastqCleaner: an interactive Bioconductor application for quality-control, filtering and trimming of FASTQ files
Source: BMC Bioinformatics. 2019 Jun 28;20:361. doi: 10.1186/s12859-019-2961-8 (PMC6599294; doi:10.1186/s12859-019-2961-8)
Supplement: Supplementary file 3 — Source code of FastqCleaner. (GZ 3273 kb) [file 12859_2019_2961_MOESM3_ESM.gz › FastqCleaner/inst/application/www/help/docs/reference/fixed_filter.html]

Remove a fixed number of bases of a ShortReadQ object from 3' or 5' — fixed\_filter • FastqCleaner


FastqCleaner
0.99.28

- Reference
- Articles
  - An Introduction to FastqCleaner

# Remove a fixed number of bases of a ShortReadQ object from 3' or 5'

`fixed_filter.Rd`

The program removes a given number of bases from the 3' or 5'
regions of the sequences contained in a ShortReadQ object

```
fixed_filter(input, trim3 = NA, trim5 = NA)
```

## Arguments

| input | `ShortReadQ` object |
| trim3 | Number of bases to remove from 3' |
| trim5 | Number of bases to remove from 5' |

## Value

Filtered `ShortReadQ`
object

## Examples

```
require('Biostrings')
require('ShortRead')

# create 6 sequences of width 20

set.seed(10)
input <- random_seq(6, 20)

# create qualities of width 20 

set.seed(10)
input_q <- random_qual(c(30,40), slength = 6, swidth = 20,
encod = 'Sanger')


# create names
input_names <- seq_names(6)

# create ShortReadQ object
my_read <- ShortReadQ(sread = input, quality = input_q, id = input_names)

# apply the filter 
filtered3 <- fixed_filter(my_read, trim5 = 5)

filtered5 <- fixed_filter(my_read, trim3 = 5)

filtered3and5 <- fixed_filter(my_read, trim3 = 10, trim5 = 5)

# look at the trimmed sequences
sread(filtered3)


#>   A DNAStringSet instance of length 6
#>     width seq
#> [1]    15 TGGTCCGGTGTTCTG
#> [2]    15 ATAGGTACAGTCCAG
#> [3]    15 GCCTCCCGCAGACGC
#> [4]    15 CCGGAATGCCCTTTC
#> [5]    15 AGCTCCAGCCGTTTG
#> [6]    15 GCGGAAAGTGAACTT


sread(filtered5)


#>   A DNAStringSet instance of length 6
#>     width seq
#> [1]    15 CGGTGTTCTGGCGGA
#> [2]    15 TACAGTCCAGTAATT
#> [3]    15 CCGCAGACGCTGGGT
#> [4]    15 ATGCCCTTTCTGAGC
#> [5]    15 CAGCCGTTTGACTTC
#> [6]    15 AAGTGAACTTAGATT


sread(filtered3and5)


#>   A DNAStringSet instance of length 6
#>     width seq
#> [1]     5 TTCTG
#> [2]     5 TCCAG
#> [3]     5 GACGC
#> [4]     5 CTTTC
#> [5]     5 GTTTG
#> [6]     5 AACTT
```

## Contents

- Arguments
- Value
- Examples

## Author

Leandro Roser learoser@gmail.com

Developed by Leandro Roser, Fernán Agüero, Daniel Sánchez.

Site built with pkgdown.
