# Supplementary material for: FastqCleaner: an interactive Bioconductor application for quality-control, filtering and trimming of FASTQ files
Source: BMC Bioinformatics. 2019 Jun 28;20:361. doi: 10.1186/s12859-019-2961-8 (PMC6599294; doi:10.1186/s12859-019-2961-8)
Supplement: Supplementary file 3 — Source code of FastqCleaner. (GZ 3273 kb) [file 12859_2019_2961_MOESM3_ESM.gz › FastqCleaner/inst/application/www/help/docs/reference/index.html]

Function reference • FastqCleaner


FastqCleaner
0.99.28

- Reference

# Reference

| All functions | |
| --- | --- |
| `adapter_filter()` | Remove full and partial adapters from a ShortReadQ object |
| `check_encoding()` | Check quality encoding |
| `complex_filter()` | Remove sequences with low complexity |
| `fixed_filter()` | Remove a fixed number of bases of a ShortReadQ object from 3' or 5' |
| `inject_letter_random()` | Inject a letter in a set of sequences at random positions |
| `launch_fqc()` | Launch FastqCleaner application |
| `length_filter()` | Filter sequences of a FASTQ file by length |
| `n_filter()` | Remove sequences with non-identified bases (Ns) from a ShortReadQ object |
| `qmean_filter()` | Filter sequences by their average quality |
| `random_length()` | Create a named object with random sequences and qualities |
| `random_qual()` | Create random qualities for a given encoding |
| `random_seq()` | Create random sequences |
| `seq_filter()` | Remove a set of sequences |
| `seq_names()` | Create sequences names |
| `trim3q_filter()` | Filter sequences with low quality in 3' tails |
| `unique_filter()` | Remove duplicated sequences in a FASTQ file |

## Contents

- All functions

Developed by Leandro Roser, Fernán Agüero, Daniel Sánchez.

Site built with pkgdown.
