# Supplementary material for: FastqCleaner: an interactive Bioconductor application for quality-control, filtering and trimming of FASTQ files
Source: BMC Bioinformatics. 2019 Jun 28;20:361. doi: 10.1186/s12859-019-2961-8 (PMC6599294; doi:10.1186/s12859-019-2961-8)
Supplement: Supplementary file 3 — Source code of FastqCleaner. (GZ 3273 kb) [file 12859_2019_2961_MOESM3_ESM.gz › FastqCleaner/inst/application/www/help/docs/reference/inject_letter_random.html]

Inject a letter in a set of sequences at random positions — inject\_letter\_random • FastqCleaner


FastqCleaner
0.99.28

- Reference
- Articles
  - An Introduction to FastqCleaner

# Inject a letter in a set of sequences at random positions

`inject_letter_random.Rd`

Inject a letter in a set of sequences at random positions

```
inject_letter_random(my_seq, how_many_seqs = NULL, how_many_letters = NULL,
  letter = "N")
```

## Arguments

| my\_seq | character vector with sequences to inject |
| how\_many\_seqs | How many sequences pick to inject Ns. An interval [min\_s, max\_s] with min\_s minimum and max\_s maximum sequences can be passed. In this case, a value is picked from the interval. If NULL, a random value within the interval [1, length(my\_seq)] is picked. |
| how\_many\_letters | How many times inject the letter in the i sequences that are going to be injected. An interval [min\_i max\_i] can be passed. In this case, a value is randomly picked for each sequence i. This value represents the number of times that the letter will be injected in the sequence i. If NULL, a random value within the interval [1, width(my\_seq[i])] is picked for each sequence i. |
| letter | Letter to inject. Default: 'N' |

## Value

character vector

## Examples

```
# For reproducible examples, make a call to set.seed before 
# running each random function

set.seed(10)
s <- random_seq(slength = 10, swidth = 20)

set.seed(10)
s <- inject_letter_random(s, how_many_seqs = 1:30, how_many= 2:10)
```

## Contents

- Arguments
- Value
- Examples

## Author

Leandro Roser learoser@gmail.com

Developed by Leandro Roser, Fernán Agüero, Daniel Sánchez.

Site built with pkgdown.
