# Supplementary material for: FastqCleaner: an interactive Bioconductor application for quality-control, filtering and trimming of FASTQ files
Source: BMC Bioinformatics. 2019 Jun 28;20:361. doi: 10.1186/s12859-019-2961-8 (PMC6599294; doi:10.1186/s12859-019-2961-8)
Supplement: Supplementary file 3 — Source code of FastqCleaner. (GZ 3273 kb) [file 12859_2019_2961_MOESM3_ESM.gz › FastqCleaner/inst/application/www/help/docs/reference/launch_fqc.html]

Launch FastqCleaner application — launch\_fqc • FastqCleaner


FastqCleaner
0.99.28

- Reference
- Articles
  - An Introduction to FastqCleaner

# Launch FastqCleaner application

`launch_fqc.Rd`

Launch FastqCleaner application

```
launch_fqc(launch.browser = TRUE, ...)
```

## Arguments

| launch.browser | Launch in browser? Default TRUE |
| ... | Additional parameters passed to `runApp` |

## Value

Launch the application, without return value

## Examples

```
# Uncomment and paste in te console to launch the application:
# launch_fqc() 

NULL


#> NULL
```

## Contents

- Arguments
- Value
- Examples

## Author

Leandro Roser learoser@gmail.com

Developed by Leandro Roser, Fernán Agüero, Daniel Sánchez.

Site built with pkgdown.
