# Supplementary material for: FastqCleaner: an interactive Bioconductor application for quality-control, filtering and trimming of FASTQ files
Source: BMC Bioinformatics. 2019 Jun 28;20:361. doi: 10.1186/s12859-019-2961-8 (PMC6599294; doi:10.1186/s12859-019-2961-8)
Supplement: Supplementary file 3 — Source code of FastqCleaner. (GZ 3273 kb) [file 12859_2019_2961_MOESM3_ESM.gz › FastqCleaner/inst/application/www/help/docs/reference/length_filter.html]

Filter sequences of a FASTQ file by length — length\_filter • FastqCleaner


FastqCleaner
0.99.28

- Reference
- Articles
  - An Introduction to FastqCleaner

# Filter sequences of a FASTQ file by length

`length_filter.Rd`

The program removes from a ShortReadQ object those sequences
with a length lower than rm.min or/and higher than rm.max

```
length_filter(input, rm.min = NA, rm.max = NA)
```

## Arguments

| input | `ShortReadQ` object |
| rm.min | Threshold value for the minimun number of bases |
| rm.max | Threshold value for the maximum number of bases |

## Value

Filtered `ShortReadQ`
object

## Examples

```
require('Biostrings')
require('ShortRead')

# create  ShortReadQ object width widths between 1 and 100

input <- random_length(100, widths = 1:100, seed = 10)


#> Error in random_length(100, widths = 1:100, seed = 10): unused argument (seed = 10)


# apply the filter, removing sequences with  10> length > 80
filtered <- length_filter(input, rm.min = 10, rm.max = 80)


#> Error in length_filter(input, rm.min = 10, rm.max = 80): objeto 'input' no encontrado


# look at the filtered sequences
sread(filtered)


#> Error in sread(filtered): objeto 'filtered' no encontrado
```

## Contents

- Arguments
- Value
- Examples

## Author

Leandro Roser learoser@gmail.com

Developed by Leandro Roser, Fernán Agüero, Daniel Sánchez.

Site built with pkgdown.
