# Supplementary material for: FastqCleaner: an interactive Bioconductor application for quality-control, filtering and trimming of FASTQ files
Source: BMC Bioinformatics. 2019 Jun 28;20:361. doi: 10.1186/s12859-019-2961-8 (PMC6599294; doi:10.1186/s12859-019-2961-8)
Supplement: Supplementary file 3 — Source code of FastqCleaner. (GZ 3273 kb) [file 12859_2019_2961_MOESM3_ESM.gz › FastqCleaner/inst/application/www/help/docs/reference/matching.html]

Remove left and right full and partial patterns — cutRseq • FastqCleaner


FastqCleaner
0.99.28

- Reference
- Articles
  - An Introduction to FastqCleaner

# Remove left and right full and partial patterns

`matching.Rd`

This set of programs are internal,
and the function adapter\_filter is recommended for trimming.
The programs can remove adapters and partial
adapters from 3' and 5'. The adapters can be anchored or not.
When indels are allowed, the error rate consists in the edit distance.
IUPAC simbols are allowed. The methods use the
`isMatchingStartingAt` function
of the Biostrings package to find matches. IUPAC symbols
are allowed in all the cases. The present function
also removes partial adapters, without the need of additional steps
(for example, creating a padded adapter with 'Ns', etc).
A similar result to the output of `trimLRPatterns`
can be obtained with the option anchored = TRUE.
When several matches are found, the function removes the subsequence
that starts in the first match when cutRseq is used, or ends
in the last match when cutLseq is used.

```
cutRseq(subject, Rpattern, with.indels = FALSE, fixed = "subject",
  method = c("exact", "er"), error_rate = 0.2, anchored = TRUE,
  ranges = FALSE, checks = TRUE, min_match_flank = 1L, ...)
```

## Arguments

| subject | `DNAString` or `DNAStringSet` object |
| Rpattern | 3' pattern, `DNAString` object |
| with.indels | Allow indels? This feature is only available for er method. |
| fixed | Parameter passed to codeisMatchingStartingAt and `isMatchingEndingAt` Default 'subject', ambiguities in the pattern only are interpreted as wildcard. See the argument fixed in codeisMatchingStartingAt and `isMatchingEndingAt` |
| method | Method used for trimming. If 'exact' the metod is based on the exact matching of the posible subsequences of the subject and the adapters. If 'er' the metod is based on the eror-rate between the subsequences, allowing mismatches. |
| error\_rate | Error rate (value in [0, 1] used for 'er' method). The error rate is the proportion of mismatches allowed between the adapter and the aligned portion of the subject. For a given adapter A, the number of allowed mismatches between each subsequence s of A and the subject is computed as: error\_rate \* L\_s, where L\_s is the length of the subsequence s. |
| anchored | Can the adapter or partial adapter be within the sequence? (anchored = FALSE) or only in the terminal regions of the sequence? (anchored = TRUE). Default TRUE (trim only flanking regions) |
| ranges | Return ranges? Default FALSE |
| checks | Perform internal checks? Default TRUE |
| min\_match\_flank | Do not trim in flanks of the subject, if a match has min\_match\_flank of less length. Default 1L (only trim with >=2 coincidences in a flank match) |
| ... | additional parameters passed to `isMatchingStartingAt` and `isMatchingEndingAt` |
| Lpattern | 5' pattern, `DNAString` object |

## Value

Edited `DNAString` or
`DNAStringSet` object

## Examples

```
# NOT RUN {
library(Biostrings)

subject <- DNAStringSet(c('ATCATGCCATCATGAT',
'CATGATATTA', 'TCATG', 'AAAAAA', 'AGGTCATG'))

Lpattern <- Rpattern <- 'TCATG'

cutLseq(subject, Lpattern)
cutLseq(subject, Lpattern, ranges = TRUE)
cutRseq(subject, Rpattern)


cutLseq(subject, Lpattern, anchored = FALSE)
cutLseq(subject, Lpattern, method = 'er', error_rate = 0.2)
cutLseq(subject, Lpattern, method = 'er', error_rate = 0.2,
with.indels = TRUE)
# }
```

## Contents

- Arguments
- Value
- Examples

## Author

Leandro Roser learoser@gmail.com

Developed by Leandro Roser, Fernán Agüero, Daniel Sánchez.

Site built with pkgdown.
