# Supplementary material for: FastqCleaner: an interactive Bioconductor application for quality-control, filtering and trimming of FASTQ files
Source: BMC Bioinformatics. 2019 Jun 28;20:361. doi: 10.1186/s12859-019-2961-8 (PMC6599294; doi:10.1186/s12859-019-2961-8)
Supplement: Supplementary file 3 — Source code of FastqCleaner. (GZ 3273 kb) [file 12859_2019_2961_MOESM3_ESM.gz › FastqCleaner/inst/application/www/help/docs/reference/n_filter.html]

Remove sequences with non-identified bases (Ns) from a ShortReadQ object — n\_filter • FastqCleaner


FastqCleaner
0.99.28

- Reference
- Articles
  - An Introduction to FastqCleaner

# Remove sequences with non-identified bases (Ns) from a ShortReadQ object

`n_filter.Rd`

This program is a wrapper to
`nFilter`.
It removes the sequences with a number of N's above
a threshold value 'rm.N'.
All the sequences with a number of N > rm.N (N >= rm.N) will be removed

```
n_filter(input, rm.N)
```

## Arguments

| input | `ShortReadQ` object |
| rm.N | Threshold value of N's to remove a sequence from the output (sequences with number of Ns > threshold are removed) For example, if rm.N is 3, all the sequences with a number of Ns > 3 (Ns >= 4) will be removed |

## Value

Filtered `ShortReadQ`
object

## Examples

```
require('Biostrings')
require('ShortRead')

# create 6 sequences of width 20
set.seed(10)
input <- random_seq(50, 20)

# inject N's
set.seed(10)
input <- inject_letter_random(input, how_many_seqs = 1:30,
how_many = 1:10)

input <- DNAStringSet(input)


# watch the N's frequency
hist(letterFrequency(input, 'N'), breaks = 0:10,
main  = 'Ns Frequency', xlab = '# Ns')


# create qualities of width 20
set.seed(10)
input_q <- random_qual(50, 20)

# create names
input_names <- seq_names(50)

# create ShortReadQ object
my_read <- ShortReadQ(sread = input, quality = input_q, id = input_names)

# apply the filter 
filtered <- n_filter(my_read, rm.N = 3)

# watch the filtered sequences
sread(filtered)


#>   A DNAStringSet instance of length 41
#>      width seq
#>  [1]    20 TGGTCCGGTGTTCTGGCGGA
#>  [2]    20 GCCTCCCGCAGACGCTGGGT
#>  [3]    20 CCGGAATGCCCTTTCTGAGC
#>  [4]    20 GCGGAAAGTGAACTTAGATT
#>  [5]    20 CGGTCCTGAAACACGGTACT
#>  ...   ... ...
#> [37]    20 ATCAATTCGTCCTGAGTTCA
#> [38]    20 AGCCCACTGGGGGAGAACGC
#> [39]    20 GCCAACGACGAAGAATCAAC
#> [40]    20 GGGAAGATCCGTTACTCTTT
#> [41]    20 NGGAATTCCCNGAANTCGCA


# watch the N's frequency
hist(letterFrequency(sread(filtered), 'N'),
main = 'Ns distribution', xlab = '')
```

## Contents

- Arguments
- Value
- Examples

## Author

Leandro Roser learoser@gmail.com

Developed by Leandro Roser, Fernán Agüero, Daniel Sánchez.

Site built with pkgdown.
