# Supplementary material for: FastqCleaner: an interactive Bioconductor application for quality-control, filtering and trimming of FASTQ files
Source: BMC Bioinformatics. 2019 Jun 28;20:361. doi: 10.1186/s12859-019-2961-8 (PMC6599294; doi:10.1186/s12859-019-2961-8)
Supplement: Supplementary file 3 — Source code of FastqCleaner. (GZ 3273 kb) [file 12859_2019_2961_MOESM3_ESM.gz › FastqCleaner/inst/application/www/help/docs/reference/plotI.html]

plotI — plotI • FastqCleaner


FastqCleaner
0.99.28

- Reference
- Articles
  - An Introduction to FastqCleaner

# plotI

`plotI.Rd`

plotI

```
plotI(x, nplots = 1, theFile = c("input", "output"), sampleSize)
```

## Value

Read ocurrence distribution plot

## Contents

- Value

Developed by Leandro Roser, Fernán Agüero, Daniel Sánchez.

Site built with pkgdown.
