# Supplementary material for: FastqCleaner: an interactive Bioconductor application for quality-control, filtering and trimming of FASTQ files
Source: BMC Bioinformatics. 2019 Jun 28;20:361. doi: 10.1186/s12859-019-2961-8 (PMC6599294; doi:10.1186/s12859-019-2961-8)
Supplement: Supplementary file 3 — Source code of FastqCleaner. (GZ 3273 kb) [file 12859_2019_2961_MOESM3_ESM.gz › FastqCleaner/inst/application/www/help/docs/reference/plotObjects.html]

plotObjects
Create the information required to construct the plots.
This is the input of myplot, which uses the values created for
this function to construct the plots — plotObjects • FastqCleaner


FastqCleaner
0.99.28

- Reference
- Articles
  - An Introduction to FastqCleaner

# plotObjects Create the information required to construct the plots. This is the input of myplot, which uses the values created for this function to construct the plots

`plotObjects.Rd`

plotObjects
Create the information required to construct the plots.
This is the input of myplot, which uses the values created for
this function to construct the plots

```
plotObjects(fq, klength, basename, maxFreq, sampleSize)
```

## Value

List with information to construct the diagnostic plots

## Contents

- Value

Developed by Leandro Roser, Fernán Agüero, Daniel Sánchez.

Site built with pkgdown.
