# Supplementary material for: FastqCleaner: an interactive Bioconductor application for quality-control, filtering and trimming of FASTQ files
Source: BMC Bioinformatics. 2019 Jun 28;20:361. doi: 10.1186/s12859-019-2961-8 (PMC6599294; doi:10.1186/s12859-019-2961-8)
Supplement: Supplementary file 3 — Source code of FastqCleaner. (GZ 3273 kb) [file 12859_2019_2961_MOESM3_ESM.gz › FastqCleaner/inst/application/www/help/docs/reference/qmean_filter.html]

Filter sequences by their average quality — qmean\_filter • FastqCleaner


FastqCleaner
0.99.28

- Reference
- Articles
  - An Introduction to FastqCleaner

# Filter sequences by their average quality

`qmean_filter.Rd`

The program removes the sequences with a quality
lower the 'minq' threshold

```
qmean_filter(input, minq, q_format = NULL, check.encod = TRUE)
```

## Arguments

| input | `ShortReadQ` object |
| minq | Quality threshold |
| q\_format | Quality format used for the file, as returned by check.encoding |
| check.encod | Check the encoding of the sequence? This argument is incompatible with q\_format |

## Value

Filtered `ShortReadQ`
object

## Examples

```
require(ShortRead)

set.seed(10)
# create 6 sequences of width 20
input <- random_seq(30, 20)

# create qualities of width 20 
## high quality (15 sequences)
set.seed(10)
my_qual <- random_qual(c(30,40), slength = 15,
                       seed = 10, encod = 'Sanger')


#> Error in random_qual(c(30, 40), slength = 15, seed = 10, encod = "Sanger"): unused argument (seed = 10)


## low quality (15 sequences)
set.seed(10)
my_qual_2 <-   random_qual(c(5,30), slength = 15,
                           seed = 10, encod = 'Sanger')


#> Error in random_qual(c(5, 30), slength = 15, seed = 10, encod = "Sanger"): unused argument (seed = 10)


# concatenate vectors
input_q<- c(my_qual, my_qual_2)


#> Error in eval(expr, envir, enclos): objeto 'my_qual' no encontrado


# create names
input_names <- seq_names(30)

# create ShortReadQ object
my_read <- ShortReadQ(sread = input, quality = input_q, id = input_names)


#> Error in ShortReadQ(sread = input, quality = input_q, id = input_names): objeto 'input_q' no encontrado


# watch the average qualities
alphabetScore(my_read) / width(my_read)


#> Error in alphabetScore(my_read): objeto 'my_read' no encontrado


# apply the filter
filtered <- qmean_filter(my_read, minq = 30)


#> Error in sread(input): objeto 'my_read' no encontrado


# watch the average qualities
alphabetScore(my_read) / width(my_read)


#> Error in alphabetScore(my_read): objeto 'my_read' no encontrado


# watch the filtered sequences
sread(filtered)


#> Error in sread(filtered): objeto 'filtered' no encontrado
```

## Contents

- Arguments
- Value
- Examples

## Author

Leandro Roser learoser@gmail.com

Developed by Leandro Roser, Fernán Agüero, Daniel Sánchez.

Site built with pkgdown.
