# Supplementary material for: FastqCleaner: an interactive Bioconductor application for quality-control, filtering and trimming of FASTQ files
Source: BMC Bioinformatics. 2019 Jun 28;20:361. doi: 10.1186/s12859-019-2961-8 (PMC6599294; doi:10.1186/s12859-019-2961-8)
Supplement: Supplementary file 3 — Source code of FastqCleaner. (GZ 3273 kb) [file 12859_2019_2961_MOESM3_ESM.gz › FastqCleaner/inst/application/www/help/docs/reference/random_length.html]

Create a named object with random sequences and qualities — random\_length • FastqCleaner


FastqCleaner
0.99.28

- Reference
- Articles
  - An Introduction to FastqCleaner

# Create a named object with random sequences and qualities

`random_length.Rd`

Create a `ShortReadQ`
object with random sequences and qualities

```
random_length(n, widths, random_widths = TRUE, replace = TRUE,
  len_prob = NULL, seq_prob = c(0.25, 0.25, 0.25, 0.25), q_prob = NULL,
  nuc = c("DNA", "RNA"), qual = NULL, encod = c("Sanger", "Illumina1.8",
  "Illumina1.5", "Illumina1.3", "Solexa"), base_name = "s", sep = "_")
```

## Arguments

| n | number of sequences |
| widths | width of the sequences |
| random\_widths | width must be picked at random from the passed parameter 'widths', considering the value as an interval where any integer can be picked. Default TRUE. Otherwise, widths are picked only from the vector passed. |
| replace | sample widths with replacement? Default TRUE. |
| len\_prob | vector with probabilities for each width value. Default NULL (equiprobability) |
| seq\_prob | a vector of four probabilities values to set the frequency of the nucleotides 'A', 'C', 'G', 'T', for DNA, or 'A', 'C', 'G', 'U', for RNA. For example = c(0.25, 0.25, 0.5, 0). Default is = c(0.25, 0.25, 0.25, 0.25) (equiprobability for the 4 bases). If the sum of the probabilities is > 1, the values will be nomalized to the range [0, 1]. |
| q\_prob | a vector of range = range(qual), with probabilities to set the frequency of each quality value. Default is equiprobability. If the sum of the probabilities is > 1, the values will be nomalized to the range [0, 1]. |
| nuc | create sequences of DNA (nucleotides = c('A', 'C', 'G', 'T')) or RNA (nucleotides = c('A, 'C', 'G', 'U'))?. Default: 'DNA' |
| qual | quality range for the sequences. It must be a range included in the selected encoding:  'Sanger' = [0, 40]  'Illumina1.8' = [0, 41]  'Illumina1.5' = [0, 40]  'Illumina1.3' = [3, 40]  'Solexa' = [-5, 40]  example: for a range from 20 to 30 in Sanger encoding, pass the argument = c(20, 30) |
| encod | sequence encoding |
| base\_name | Base name for strings |
| sep | Character separing base names and the read number. Default: '\_' |

## Value

`ShortReadQ` object

## Examples

```
# For reproducible examples, make a call to set.seed before 
# running each random function

set.seed(10)
s1 <- random_seq(slength = 10, swidth = 20)
s1


#>   A DNAStringSet instance of length 10
#>      width seq
#>  [1]    20 TGGTCCGGTGTTCTGGCGGA
#>  [2]    20 ATAGGTACAGTCCAGTAATT
#>  [3]    20 GCCTCCCGCAGACGCTGGGT
#>  [4]    20 CCGGAATGCCCTTTCTGAGC
#>  [5]    20 AGCTCCAGCCGTTTGACTTC
#>  [6]    20 GCGGAAAGTGAACTTAGATT
#>  [7]    20 CGGTCCTGAAACACGGTACT
#>  [8]    20 TCCACAGTCAACCCGCCGAC
#>  [9]    20 TTGGAGAATTTATTAGCCGG
#> [10]    20 GCGGTTATTCCCCTAGTGAT


set.seed(10)
s2 <- random_seq(slength = 10, swidth = 20,
prob = c(0.6, 0.1, 0.3, 0))
s2


#>   A DNAStringSet instance of length 10
#>      width seq
#>  [1]    20 AAAGAAAAGAGAAAAAAAAG
#>  [2]    20 GGGAAGGAGAAAAGAGGCGA
#>  [3]    20 AAAGAAAAAGACAAAAAAAA
#>  [4]    20 AAAAGGAAAAAAGAAAACAA
#>  [5]    20 GAAGAAGAAAAGAAAGAAGA
#>  [6]    20 AAAACGCAGAGGAAGCAGGG
#>  [7]    20 AAAAAAGAGCGAGAAAGGAA
#>  [8]    20 GAACAGAGACGAAAAAAACA
#>  [9]    20 GAAAGAGCGAAGGGCAAAAA
#> [10]    20 AAAAAGGGGAAAAAGAAAGG
```

## Contents

- Arguments
- Value
- Examples

## Author

Leandro Roser learoser@gmail.com

Developed by Leandro Roser, Fernán Agüero, Daniel Sánchez.

Site built with pkgdown.
