# Supplementary material for: FastqCleaner: an interactive Bioconductor application for quality-control, filtering and trimming of FASTQ files
Source: BMC Bioinformatics. 2019 Jun 28;20:361. doi: 10.1186/s12859-019-2961-8 (PMC6599294; doi:10.1186/s12859-019-2961-8)
Supplement: Supplementary file 3 — Source code of FastqCleaner. (GZ 3273 kb) [file 12859_2019_2961_MOESM3_ESM.gz › FastqCleaner/inst/application/www/help/docs/reference/random_qual.html]

Create random qualities for a given encoding — random\_qual • FastqCleaner


FastqCleaner
0.99.28

- Reference
- Articles
  - An Introduction to FastqCleaner

# Create random qualities for a given encoding

`random_qual.Rd`

Create a `BStringSet`
object
with random qualities

```
random_qual(slength, swidth, qual = NULL, encod = c("Sanger", "Illumina1.8",
  "Illumina1.5", "Illumina1.3", "Solexa"), prob = NULL)
```

## Arguments

| slength | number of sequences |
| swidth | width of the sequences |
| qual | quality range for the sequences. It must be a range included in the selected encoding:  'Sanger' = [0, 40]  'Illumina1.8' = [0, 41]  'Illumina1.5' = [0, 40]  'Illumina1.3' = [3, 40]  'Solexa' = [-5, 40]  example: for a range from 20 to 30 in Sanger encoding, pass the argument = c(20, 30) |
| encod | sequence encoding |
| prob | a vector of range = range(qual), with probabilities to set the frequency of each quality value. Default is equiprobability. If the sum of the probabilities is > 1, the values will be nomalized to the range [0, 1]. |

## Value

`BStringSet` object

## Examples

```
q <- random_qual(30, 20)
q


#>   A BStringSet instance of length 30
#>      width seq
#>  [1]    20 56F)!=<.+##1#=)0FA,3
#>  [2]    20 4F6&/#H#!%">#802<(&8
#>  [3]    20 =-F(-?A-?<2#+1F+H>G0
#>  [4]    20 4(CA6HBF1#5?D<%H(96?
#>  [5]    20 H7+CD8:)5<))=.G27>&@
#>  ...   ... ...
#> [26]    20 )-BA*%D6!-4<4+%1=+D#
#> [27]    20 267I0?;H:>!:03EB+87!
#> [28]    20 =00-9'F*$I*.>/F+;#9A
#> [29]    20 =%#>FH<H5#$B=2,7G>#=
#> [30]    20 9>+%*/,8I$")$?@!C,A,
```

## Contents

- Arguments
- Value
- Examples

## Author

Leandro Roser learoser@gmail.com

Developed by Leandro Roser, Fernán Agüero, Daniel Sánchez.

Site built with pkgdown.
