# Supplementary material for: FastqCleaner: an interactive Bioconductor application for quality-control, filtering and trimming of FASTQ files
Source: BMC Bioinformatics. 2019 Jun 28;20:361. doi: 10.1186/s12859-019-2961-8 (PMC6599294; doi:10.1186/s12859-019-2961-8)
Supplement: Supplementary file 3 — Source code of FastqCleaner. (GZ 3273 kb) [file 12859_2019_2961_MOESM3_ESM.gz › FastqCleaner/inst/application/www/help/docs/reference/random_seq.html]

Create random sequences — random\_seq • FastqCleaner


FastqCleaner
0.99.28

- Reference
- Articles
  - An Introduction to FastqCleaner

# Create random sequences

`random_seq.Rd`

Create a
`DNAStringSet` object
with random sequences

```
random_seq(slength, swidth, nuc = c("DNA", "RNA"), prob = c(0.25, 0.25,
  0.25, 0.25))
```

## Arguments

| slength | Number of sequences |
| swidth | Width of the sequences |
| nuc | Create sequences of DNA (nucleotides = c('A', 'C', 'G', 'T')) or RNA (nucleotides = c('A, 'C', 'G', 'U'))?. Default: 'DNA' |
| prob | A vector of four probability values used to set the frequency of the nucleotides 'A', 'C', 'G', 'T', for DNA, or 'A', 'C', 'G', 'U', for RNA. For example = c(0.25, 0.25, 0.5, 0). Default is = c(0.25, 0.25, 0.25, 0.25) (equiprobability for the 4 bases). If the sum of the probabilities is > 1, the values will be nomalized to the range [0, 1]. |

## Value

`DNAStringSet` object

## Examples

```
# For reproducible examples, make a call to set.seed before 
# running each random function

set.seed(10)
s1 <- random_seq(slength = 10, swidth = 20)
s1


#>   A DNAStringSet instance of length 10
#>      width seq
#>  [1]    20 TGGTCCGGTGTTCTGGCGGA
#>  [2]    20 ATAGGTACAGTCCAGTAATT
#>  [3]    20 GCCTCCCGCAGACGCTGGGT
#>  [4]    20 CCGGAATGCCCTTTCTGAGC
#>  [5]    20 AGCTCCAGCCGTTTGACTTC
#>  [6]    20 GCGGAAAGTGAACTTAGATT
#>  [7]    20 CGGTCCTGAAACACGGTACT
#>  [8]    20 TCCACAGTCAACCCGCCGAC
#>  [9]    20 TTGGAGAATTTATTAGCCGG
#> [10]    20 GCGGTTATTCCCCTAGTGAT


set.seed(10)
s2 <- random_seq(slength = 10, swidth = 20,
prob = c(0.6, 0.1, 0.3, 0))
s2


#>   A DNAStringSet instance of length 10
#>      width seq
#>  [1]    20 AAAGAAAAGAGAAAAAAAAG
#>  [2]    20 GGGAAGGAGAAAAGAGGCGA
#>  [3]    20 AAAGAAAAAGACAAAAAAAA
#>  [4]    20 AAAAGGAAAAAAGAAAACAA
#>  [5]    20 GAAGAAGAAAAGAAAGAAGA
#>  [6]    20 AAAACGCAGAGGAAGCAGGG
#>  [7]    20 AAAAAAGAGCGAGAAAGGAA
#>  [8]    20 GAACAGAGACGAAAAAAACA
#>  [9]    20 GAAAGAGCGAAGGGCAAAAA
#> [10]    20 AAAAAGGGGAAAAAGAAAGG
```

## Contents

- Arguments
- Value
- Examples

## Author

Leandro Roser learoser@gmail.com

Developed by Leandro Roser, Fernán Agüero, Daniel Sánchez.

Site built with pkgdown.
