# Supplementary material for: FastqCleaner: an interactive Bioconductor application for quality-control, filtering and trimming of FASTQ files
Source: BMC Bioinformatics. 2019 Jun 28;20:361. doi: 10.1186/s12859-019-2961-8 (PMC6599294; doi:10.1186/s12859-019-2961-8)
Supplement: Supplementary file 3 — Source code of FastqCleaner. (GZ 3273 kb) [file 12859_2019_2961_MOESM3_ESM.gz › FastqCleaner/inst/application/www/help/docs/reference/seq_filter.html]

Remove a set of sequences — seq\_filter • FastqCleaner


FastqCleaner
0.99.28

- Reference
- Articles
  - An Introduction to FastqCleaner

# Remove a set of sequences

`seq_filter.Rd`

Removes a set of sequences

```
seq_filter(input, rm.seq)
```

## Arguments

| input | `ShortReadQ` object |
| rm.seq | Ccharacter vector with sequences to remove |

## Value

Filtered `ShortReadQ`
object

## Examples

```
require(ShortRead)

set.seed(10)
input <- random_length(30, 3:7)
rm.seq  = c('TGGTC', 'CGGT', 'GTTCT', 'ATA')

# verify that some sequences match
match_before <- unlist(lapply(rm.seq,
 function(x) grep(x, as.character(sread(input)))))

filtered <- seq_filter(input,rm.seq =  rm.seq)

# verify that matching sequences were removed
match_after <- unlist(lapply(rm.seq,
function(x) grep(x, as.character(sread(filtered)))))
```

## Contents

- Arguments
- Value
- Examples

## Author

Leandro Roser learoser@gmail.com

Developed by Leandro Roser, Fernán Agüero, Daniel Sánchez.

Site built with pkgdown.
