# Supplementary material for: FastqCleaner: an interactive Bioconductor application for quality-control, filtering and trimming of FASTQ files
Source: BMC Bioinformatics. 2019 Jun 28;20:361. doi: 10.1186/s12859-019-2961-8 (PMC6599294; doi:10.1186/s12859-019-2961-8)
Supplement: Supplementary file 3 — Source code of FastqCleaner. (GZ 3273 kb) [file 12859_2019_2961_MOESM3_ESM.gz › FastqCleaner/inst/application/www/help/docs/reference/seq_names.html]

Create sequences names — seq\_names • FastqCleaner


FastqCleaner
0.99.28

- Reference
- Articles
  - An Introduction to FastqCleaner

# Create sequences names

`seq_names.Rd`

Create `BStringSet`
object with names

```
seq_names(n, base_name = "s", sep = "_")
```

## Arguments

| n | Number of reads |
| base\_name | Base name for strings |
| sep | Character separing base names and the read number. Default: '\_ |

## Value

`BStringSet` object

## Examples

```
snames <- seq_names(10)
snames


#>   A BStringSet instance of length 10
#>      width seq
#>  [1]     3 s_1
#>  [2]     3 s_2
#>  [3]     3 s_3
#>  [4]     3 s_4
#>  [5]     3 s_5
#>  [6]     3 s_6
#>  [7]     3 s_7
#>  [8]     3 s_8
#>  [9]     3 s_9
#> [10]     4 s_10


snames2 <- seq_names(10, base_name = 's', sep = '.')
snames2


#>   A BStringSet instance of length 10
#>      width seq
#>  [1]     3 s.1
#>  [2]     3 s.2
#>  [3]     3 s.3
#>  [4]     3 s.4
#>  [5]     3 s.5
#>  [6]     3 s.6
#>  [7]     3 s.7
#>  [8]     3 s.8
#>  [9]     3 s.9
#> [10]     4 s.10
```

## Contents

- Arguments
- Value
- Examples

Developed by Leandro Roser, Fernán Agüero, Daniel Sánchez.

Site built with pkgdown.
