# Supplementary material for: FastqCleaner: an interactive Bioconductor application for quality-control, filtering and trimming of FASTQ files
Source: BMC Bioinformatics. 2019 Jun 28;20:361. doi: 10.1186/s12859-019-2961-8 (PMC6599294; doi:10.1186/s12859-019-2961-8)
Supplement: Supplementary file 3 — Source code of FastqCleaner. (GZ 3273 kb) [file 12859_2019_2961_MOESM3_ESM.gz › FastqCleaner/inst/application/www/help/docs/reference/trim3q_filter.html]

Filter sequences with low quality in 3' tails — trim3q\_filter • FastqCleaner


FastqCleaner
0.99.28

- Reference
- Articles
  - An Introduction to FastqCleaner

# Filter sequences with low quality in 3' tails

`trim3q_filter.Rd`

The program removes from the 3' tails of the sequences
a set of nucleotides showing a quality < a threshold value in a
ShortReadQ object

```
trim3q_filter(input, rm.3qual, q_format = NULL, check.encod = TRUE,
  remove_zero = TRUE)
```

## Arguments

| input | `ShortReadQ` object |
| rm.3qual | Quality threshold for 3' tails |
| q\_format | Quality format used for the file, as returned by check\_encoding |
| check.encod | Check the encoding of the sequence? This argument is incompatible with q\_format. Default TRUE |
| remove\_zero | Remove zero-length sequences? |

## Value

Filtered `ShortReadQ`
object

## Examples

```
require('Biostrings')
require('ShortRead')

# create 6 sequences of width 20
set.seed(10)
input <- random_seq(6, 20)

# create qualities of width 15 and paste to qualities
# of length 5 used for the tails.
# for two of the sequences, put low qualities in tails

set.seed(10)
my_qual <- random_qual(c(30,40), slength = 6, swidth = 15,
encod = 'Sanger')

set.seed(10)
tails <-   random_qual(c(30,40), slength = 6, swidth = 5,
 encod = 'Sanger')

set.seed(10)
tails[2:3] <- random_qual(c(3, 20), slength = 2,
swidth = 5,  encod = 'Sanger')
my_qual <- paste0(my_qual, tails)
input_q <- BStringSet(my_qual)
# create names
input_names <- seq_names(6)

# create ShortReadQ object
my_read <- ShortReadQ(sread = input,
quality = input_q, id = input_names)

# apply the filter 
filtered <- trim3q_filter(my_read, rm.3qual = 28)

# look at the trimmed sequences
sread(filtered)


#>   A DNAStringSet instance of length 6
#>     width seq
#> [1]    20 TGGTCCGGTGTTCTGGCGGA
#> [2]    15 ATAGGTACAGTCCAG
#> [3]    15 GCCTCCCGCAGACGC
#> [4]    20 CCGGAATGCCCTTTCTGAGC
#> [5]    20 AGCTCCAGCCGTTTGACTTC
#> [6]    20 GCGGAAAGTGAACTTAGATT
```

## Contents

- Arguments
- Value
- Examples

## Author

Leandro Roser learoser@gmail.com

Developed by Leandro Roser, Fernán Agüero, Daniel Sánchez.

Site built with pkgdown.
