# Supplementary material for: FastqCleaner: an interactive Bioconductor application for quality-control, filtering and trimming of FASTQ files
Source: BMC Bioinformatics. 2019 Jun 28;20:361. doi: 10.1186/s12859-019-2961-8 (PMC6599294; doi:10.1186/s12859-019-2961-8)
Supplement: Supplementary file 3 — Source code of FastqCleaner. (GZ 3273 kb) [file 12859_2019_2961_MOESM3_ESM.gz › FastqCleaner/inst/application/www/help/docs/reference/unique_filter.html]

Remove duplicated sequences in a FASTQ file — unique\_filter • FastqCleaner


FastqCleaner
0.99.28

- Reference
- Articles
  - An Introduction to FastqCleaner

# Remove duplicated sequences in a FASTQ file

`unique_filter.Rd`

This program is a wrapper to
`occurrenceFilter`.
It removes the duplicated sequences of a FASTQ file.

```
unique_filter(input)
```

## Arguments

| input | `ShortReadQ` object |

## Value

Filtered `ShortReadQ` object

## Examples

```
require('Biostrings')
require('ShortRead')

set.seed(10)
s <- random_seq(10, 10)
s <- sample(s, 30, replace = TRUE)
q <- random_qual(30, 10)
n <- seq_names(30)

my_read <- ShortReadQ(sread = s, quality = q, id = n)

# check presence of duplicates
isUnique(as.character(sread(my_read)))


#>  [1] FALSE  TRUE FALSE FALSE FALSE FALSE FALSE FALSE FALSE FALSE FALSE FALSE
#> [13] FALSE FALSE FALSE FALSE FALSE FALSE FALSE FALSE FALSE FALSE FALSE FALSE
#> [25] FALSE FALSE FALSE FALSE FALSE FALSE


# apply the filter
filtered <- unique_filter(my_read)

isUnique(as.character(sread(filtered)))


#>  [1] TRUE TRUE TRUE TRUE TRUE TRUE TRUE TRUE TRUE TRUE
```

## Contents

- Arguments
- Value
- Examples

## Author

Leandro Roser learoser@gmail.com

Developed by Leandro Roser, Fernán Agüero, Daniel Sánchez.

Site built with pkgdown.
