# Supplementary material for: FastqCleaner: an interactive Bioconductor application for quality-control, filtering and trimming of FASTQ files
Source: BMC Bioinformatics. 2019 Jun 28;20:361. doi: 10.1186/s12859-019-2961-8 (PMC6599294; doi:10.1186/s12859-019-2961-8)
Supplement: Supplementary file 3 — Source code of FastqCleaner. (GZ 3273 kb) [file 12859_2019_2961_MOESM3_ESM.gz › FastqCleaner/inst/application/www/help/index.html]

An Introduction to FastqCleaner


# An Introduction to FastqCleaner

Leandro Roser1, Fernán Agüero1 and Daniel Sánchez1\*

1IIB-INTECH - UNSAM

\*learoser@gmail.com

#### *May 18, 2018*

#### Abstract

*FastqCleaner* is an interactive application
for both quality control and pre-processing of FASTQ files.
The interface, based on a Bioconductor back-end,
provides diagnostic information for the input and output
data and a series of filtering and trimming operations in
an interactive framework.

# Contents

- 1 Launching the application
- 2 Description of the application
  - 2.1 First panel
    - 2.1.1 Selecting operations
    - 2.1.2 Loading files
    - 2.1.3 Advanced options
  - 2.2 Second panel
  - 2.3 Third panel
- 3 A worked example: FASTQ processing in a nutshell
- 4 Advanced use of the package
  - 4.1 Main functions
  - 4.2 Auxiliary functions
- 5 Contact information

# 1 Launching the application

The interactive application can be launched in R with the following command:

```
library('FastqCleaner')
launch_fqc()
```

As an alternative method, an RStudio addin
(RStudio version 0.99.878 or higher required) installed with the package
can be found in the Addins menu (Figure 1).
This button allows the direct launch of the application with a single click.

**Figure 1**: addin of the app in RStudio (RStudio version >= 0.99.878 required)

# 2 Description of the application

The application contains three main panels, as described below.

## 2.1 First panel

The first panel includes two elements: a dashboard for selection
of trimming and filtering operations, and a menu for selection
of the input file/s (Fig. 2).

**Figure 2**: Panel 1, with each compontent indicated with a number.
See explanation for each element below

### 2.1.1 Selecting operations

The “operations menu” (Fig. 2, elements 1 to 8) shows
the available operations for file processing:

1. **Remove by N(s)**: removes sequences with a number of Ns
   (non identified bases) above a selected threshold value
2. **Remove low complexity sequences**: remove sequences with a value of
   complexity above a threshold value
3. **Remove adapters**: removes adapters and partial adapters.
   Adapter sequences from both ends of single or paired read reads
   can be selected. Sequences can be reverse-complemented before processing.
   The program also allows to consider indels
   and/or anchored adapters.
4. **Filter by average quality**: computes the average quality of sequences
   and removes those with a value below a given threshold
5. **Trim low quality 3’ tails**: removes the 3’ tails of sequences that
   are below a given threshold
6. **Trim 3’ or 5’ by a fixed number**: removes a fixed number of bases
   from the 3’ and/or 5’ ends in the complete set of sequences
7. **Filter sequences by length**: removes all the sequences with a number
   of bases below a threshold value
8. **Remove duplicated sequences**: removes duplicated reads, conserving
   only one copy of each sequence present in the file

### 2.1.2 Loading files

The “file selection menu” (Fig. 2, elements 9 to 17)
contains options to handle the input file (type of file, file selection),
buttons to run, clear and reset the aplication, and the “advanced” submenu:

9. **Single-end reads / paired-end reads**: type of input files
10. **“FILE” button**: to select an input file
11. **“RUN!” button**: to run the program
12. **Output format**: to select whether the output file should be compressed
    (.gz) or not
13. **“CLEAR” button**: to clear the configuration of the operations menu
    that have been selected in the first panel, but keeping the input file(s)
14. **“RESET” button**: to restart the application, removing the
    input file(s) and the selected configurations
15. **Selection notificator**: information of the path of the selected file/s
16. **Encoding notificator**: information of the input file/s encoding
17. **Advanced options button**: to select a custom encoding and set
    the number of reads included in each chunk for processing, as described below

### 2.1.3 Advanced options

The “advanced options submenu” (Fig. 3) allows to customize some fine aspects
of the trimming and filtering process:

**Figure 3**: Advanced options submenu

18. **Encoding menu**: in addition to the default approach used by the
    program (auto-detection of file encoding), users can select a standard encoding
    from a list
19. **Chunk size**: the program takes this number of reads at random from
    the file (default: 1000000), for encoding detection

## 2.2 Second panel

The second panel (“file operations” panel, Fig. 4) shows the operations
that were sucessfuly performed on the input file after running the program.

**Figure 4**: File operations panel, with its elements

The panel contains the following elements:

20. **Files location**: location of input and output files
21. **Operations performed**: operations perfomed on the input file.
    Each individual display indicates the number of reads that
    passed the corresponding filter

## 2.3 Third panel

The third panel ( “live results” panel, Fig. 5) shows
interactive diagnostics plots for both input and output files.
The program takes a random sample of reads for
construction of the plots (default: 10000 reads).

**Figure 5**: Live results panel

The panel includes the following options in the menu located on the left:

22. **Sample size**: the sample size used for construction of the plots.
    Default: 10000 reads
23. **Input / output**: show diagnostics plots for input or output files?
24. **Diagnostics plots**: the plot to be shown, that can be one of the
    following:

    - *Per cycle quality*: quality plots across reads for each cycle
      (i.e., sequence position)
    - *Per cycle mean quality*: average quality across reads per base,
      for each cycle (i.e., sequence position)
    - *Mean quality distribution*: Quality distribution, using for
      the construction of the histogram the mean quality of each read
    - *% reads with Phred scores > threshold*: % of reads with all
      the quality values > threshold
    - *Per cycle base proportion*: Proportion of each base
      (average across reads) in each cycle. It also shows the proporion of N’s
    - *CG content*: % CG and % AT (average across reads) for each cycle
    - *CG content distribution over all reads*: histogram for % reads with
      a given % CG
    - *Read length distribution*: % reads vs read length (bp)
    - *Read ocurrence distribution*: % reads that ocurr at different
      frequencies values in the file. The plot also includes a table
    - *Relative k-mer diversity*: unique k-mers / all posible
      kmers for each cycle
25. **Select k-mer size**: k-mer size for the k-mers frequency plot
26. **Top sequences in duplication level analysis**: a list of duplicated
    sequences, ordered from high to low duplication level,
    can be desplegated from the “read ocurrence distribution” plot.
    The number selected here indicates how many sequences should be shown.
    Note that the frequency of reads are relative to the sample size selected
    (i.e., fold-times in relation to those reads present only once in the sample)
27. **Plot panel**

# 3 A worked example: FASTQ processing in a nutshell

A sample FASTQ (gz-compressed) file ‘example.fastq.gz’ can
be downloaded with the following command in R:

```
download.file("https://goo.gl/hb4Kr9", "example_fastq.gz")
```

A direct download is provided in 
this link .

A tipical *FastqCleaner* workflow starts with the input file/s
upload (Fig. 6).

**Figure 6**: File input menu. The example shows a single-end reads case
(sample file ‘example.fastq.gz’). For paired-end reads, the selection of the
corresponding library type generates an additional button to upload the
second file.

The file encoding is automatically detected by the program, but it can also be
manually specified in the advanced submenu (Fig. 7). This menu also offers an
option to customize the chunk size used for processing.

**Figure 7**: Advanced submanu

Next, the operations to be performed on the input file are selected from the
operations menu (Fig. 8).

**Figure 8**: Selection of operations. A dialog box shows the input expected
for the program. To use a filter, the “Use filter?” checkbox must
be checked. A filter in use is indicated with a checkmark in the filter box

The program then starts to run after pressing the “RUN!” button (Fig. 9).

**Figure 9**: “RUN!” button action

Post-processing results are shown in the second panel (Fig. 10).

**Figure 10**: Second panel of the app, showing the operations performed and
the paths of the input and output files

The type of plot to be displayed and the options for the construction of the
plot are available in the third panel (Fig. 11). This panel also show the
selected plot/s.

**Figure 11**: Third panel, showing as example a “CG” content plot.
for the output file

To clean the operations, for example to run a different
configuration, the “CLEAN” i(Fig. 11) must be pressed. The “RESET” button
(Fig. 11) restarts the interface.

Additional help can be found in the “help” button located at the top-right
of the app (Fig. 12).

**Figure 12**: help button. A webpage with information will be open

# 4 Advanced use of the package

*FastqCleaner* separates the interface from the implementation.
In consequence, the processing functions of the package can be used
as standard functions from the command line.
Most of the functions make intensive
use of *Biostrings* and *ShortRead*. Trimming
and filtering is performed on ShortReadQ objects. A complete documentation
for the functions is available in this link

The functions included in the package are described in the following section.

## 4.1 Main functions

- **adapter\_filter**

Based on the *Biostrings* isMatchingStartingAt and
isMatchingEndingAt functions. It can remove adapters and partial adapters
from the 3’ and 5’ sequence ends. Adapters can be anchored or not.
Two methods are available: one based on the exact matching of the
sequences and the adapter, and other in a mismatch rate. For this latter,
when indels are allowed, the method is based on the “edit distance” of
the sequences.

```
### Examples
```

```
require("Biostrings")
require("ShortRead")
require("FastqCleaner")
```

```
# create sequences
set.seed(10) # nota that the use of set.seed before the call to the 
             # random generators allows reproducibility of the
             # examples
input <- random_seq(6, 43)
input
```

```
##   A DNAStringSet instance of length 6
##     width seq
## [1]    43 TGGTCCGGTGTTCTGGCGGAATAGGTACAGTCCAGTAATTGCC
## [2]    43 TCCCGCAGACGCTGGGTCCGGAATGCCCTTTCTGAGCAGCTCC
## [3]    43 AGCCGTTTGACTTCGCGGAAAGTGAACTTAGATTCGGTCCTGA
## [4]    43 AACACGGTACTTCCACAGTCAACCCGCCGACTTGGAGAATTTA
## [5]    43 TTAGCCGGGCGGTTATTCCCCTAGTGATCTTACTAAGATTTGC
## [6]    43 AATACCTAAGCGAAGTGACAGATATGTTCGTCATTCATCCAGG
```

```
# create qualities of width 50
set.seed(10)
input_q <- random_qual(c(30,40), slength = 6, swidth = 50, 
encod = "Sanger")

# create names
input_names <- seq_names(length(input))


### FULL ADAPTER IN 3'
adapter <- "ATCGACT"

# Create sequences with adapter
my_seqs <- paste0(input, adapter)
my_seqs <- DNAStringSet(my_seqs)
my_seqs
```

```
##   A DNAStringSet instance of length 6
##     width seq
## [1]    50 TGGTCCGGTGTTCTGGCGGAATAGGTACAGTCCAGTAATTGCCATCGACT
## [2]    50 TCCCGCAGACGCTGGGTCCGGAATGCCCTTTCTGAGCAGCTCCATCGACT
## [3]    50 AGCCGTTTGACTTCGCGGAAAGTGAACTTAGATTCGGTCCTGAATCGACT
## [4]    50 AACACGGTACTTCCACAGTCAACCCGCCGACTTGGAGAATTTAATCGACT
## [5]    50 TTAGCCGGGCGGTTATTCCCCTAGTGATCTTACTAAGATTTGCATCGACT
## [6]    50 AATACCTAAGCGAAGTGACAGATATGTTCGTCATTCATCCAGGATCGACT
```

```
# create ShortReadQ object
my_read <- ShortReadQ(sread = my_seqs, quality = input_q, id = input_names)

# trim adapter
filtered <- adapter_filter(my_read, Lpattern = adapter)
sread(filtered)
```

```
##   A DNAStringSet instance of length 6
##     width seq
## [1]    50 TGGTCCGGTGTTCTGGCGGAATAGGTACAGTCCAGTAATTGCCATCGACT
## [2]    50 TCCCGCAGACGCTGGGTCCGGAATGCCCTTTCTGAGCAGCTCCATCGACT
## [3]    50 AGCCGTTTGACTTCGCGGAAAGTGAACTTAGATTCGGTCCTGAATCGACT
## [4]    50 AACACGGTACTTCCACAGTCAACCCGCCGACTTGGAGAATTTAATCGACT
## [5]    50 TTAGCCGGGCGGTTATTCCCCTAGTGATCTTACTAAGATTTGCATCGACT
## [6]    50 AATACCTAAGCGAAGTGACAGATATGTTCGTCATTCATCCAGGATCGACT
```

```
### PARTIAL ADAPTER IN 5'
adapter <- "ATCGACT"
subadapter <- subseq(adapter, 1, 4)

# Create sequences with adapter
my_seqs <- paste0(input, subadapter)
my_seqs <- DNAStringSet(my_seqs)
my_seqs
```

```
##   A DNAStringSet instance of length 6
##     width seq
## [1]    47 TGGTCCGGTGTTCTGGCGGAATAGGTACAGTCCAGTAATTGCCATCG
## [2]    47 TCCCGCAGACGCTGGGTCCGGAATGCCCTTTCTGAGCAGCTCCATCG
## [3]    47 AGCCGTTTGACTTCGCGGAAAGTGAACTTAGATTCGGTCCTGAATCG
## [4]    47 AACACGGTACTTCCACAGTCAACCCGCCGACTTGGAGAATTTAATCG
## [5]    47 TTAGCCGGGCGGTTATTCCCCTAGTGATCTTACTAAGATTTGCATCG
## [6]    47 AATACCTAAGCGAAGTGACAGATATGTTCGTCATTCATCCAGGATCG
```

```
# create ShortReadQ object
my_read <- ShortReadQ(sread = my_seqs, quality = subseq(input_q, 1, 47), 
id = input_names)

# trim adapter
filtered <- adapter_filter(my_read, Rpattern = adapter)
sread(filtered)
```

```
##   A DNAStringSet instance of length 6
##     width seq
## [1]    43 TGGTCCGGTGTTCTGGCGGAATAGGTACAGTCCAGTAATTGCC
## [2]    43 TCCCGCAGACGCTGGGTCCGGAATGCCCTTTCTGAGCAGCTCC
## [3]    43 AGCCGTTTGACTTCGCGGAAAGTGAACTTAGATTCGGTCCTGA
## [4]    43 AACACGGTACTTCCACAGTCAACCCGCCGACTTGGAGAATTTA
## [5]    43 TTAGCCGGGCGGTTATTCCCCTAGTGATCTTACTAAGATTTGC
## [6]    43 AATACCTAAGCGAAGTGACAGATATGTTCGTCATTCATCCAGG
```

Documentation of the function

- **complex\_filter**

Removes low complexity sequences, computing the entropy with the
dinucleotide frequency:
\[H\_i = -\sum d\_i \* log\_2(d\_i)\]

where: \(d\_i = D\_i/ \sum\_i^n D\_i\) represents the frequency of
dinucleotides of the sequence \(i\) relative to the frequency
in the whole pool of sequences.

The relation \(H\_i/H\_r\) between \(H\_i\) and a reference entropy value \(H\_r\)
is computed, and the obtained relations are compared with a given complexity
threshold. By default the program uses a reference entropy
of 3.908, that corresponds to the entropy of the human genome in bits,
and a complexity threshold of 0.5.

```
# create  sequences of different width
set.seed(10)
input <- lapply(c(0, 6, 10, 16, 20, 26, 30, 36, 40), 
            function(x) random_seq(1, x))


# create repetitive "CG" sequences with length adequante 
# for a total length input +  CG = 40

CG <- lapply(c(20, 17, 15, 12, 10, 7, 5, 2, 0), 
            function(x) paste(rep("CG", x), collapse = ""))

# concatenate input and CG
input  <- mapply("paste", input, CG, sep = "")
input <- DNAStringSet(input)
input
```

```
##   A DNAStringSet instance of length 9
##     width seq
## [1]    40 CGCGCGCGCGCGCGCGCGCGCGCGCGCGCGCGCGCGCGCG
## [2]    40 TGGTCCCGCGCGCGCGCGCGCGCGCGCGCGCGCGCGCGCG
## [3]    40 GGTGTTCTGGCGCGCGCGCGCGCGCGCGCGCGCGCGCGCG
## [4]    40 CGGAATAGGTACAGTCCGCGCGCGCGCGCGCGCGCGCGCG
## [5]    40 CAGTAATTGCCTCCCGCAGACGCGCGCGCGCGCGCGCGCG
## [6]    40 CGCTGGGTCCGGAATGCCCTTTCTGACGCGCGCGCGCGCG
## [7]    40 GCAGCTCCAGCCGTTTGACTTCGCGGAAAGCGCGCGCGCG
## [8]    40 TGAACTTAGATTCGGTCCTGAAACACGGTACTTCCACGCG
## [9]    40 CAGTCAACCCGCCGACTTGGAGAATTTATTAGCCGGGCGG
```

```
# plot relative entropy (E, Shannon 1948)
H_plot <- function(x, H_max = 3.908135) {
    freq <- dinucleotideFrequency(x)
    freq  <- freq /rowSums(freq)
    H <- -rowSums(freq  * log2(freq), na.rm = TRUE)
    plot(H/H_max, type="l", xlab = "Sequence", ylab= "E")
    points(H/H_max, col = "#1a81c2", pch = 16, cex = 2)
}

H_plot(input)
```

**Figure 13**: Relative entropy plot for the sequences before the operation

```
# create qualities of widths 40
set.seed(10)
input_q <- random_qual(c(30,40), slength = 9, swidth = 40, 
           encod = "Sanger")

# create names
input_names <- seq_names(9)


# create ShortReadQ object
my_read <- ShortReadQ(sread = input, quality = input_q, id = input_names)

# apply the filter, 
filtered <- complex_filter(my_read)
sread(filtered)
```

```
##   A DNAStringSet instance of length 7
##     width seq
## [1]    40 GGTGTTCTGGCGCGCGCGCGCGCGCGCGCGCGCGCGCGCG
## [2]    40 CGGAATAGGTACAGTCCGCGCGCGCGCGCGCGCGCGCGCG
## [3]    40 CAGTAATTGCCTCCCGCAGACGCGCGCGCGCGCGCGCGCG
## [4]    40 CGCTGGGTCCGGAATGCCCTTTCTGACGCGCGCGCGCGCG
## [5]    40 GCAGCTCCAGCCGTTTGACTTCGCGGAAAGCGCGCGCGCG
## [6]    40 TGAACTTAGATTCGGTCCTGAAACACGGTACTTCCACGCG
## [7]    40 CAGTCAACCCGCCGACTTGGAGAATTTATTAGCCGGGCGG
```

```
H_plot(sread(filtered))
```

**Figure 14**: Relative entropy plot for the sequences after the operation

Documentation of the function

- **fixed\_filter**

Removes the specified number of bases from 3’ or 5’.

```
# create sequences, qualities and names of width 20
set.seed(10)
input <- random_seq(6, 20)
input
```

```
##   A DNAStringSet instance of length 6
##     width seq
## [1]    20 TGGTCCGGTGTTCTGGCGGA
## [2]    20 ATAGGTACAGTCCAGTAATT
## [3]    20 GCCTCCCGCAGACGCTGGGT
## [4]    20 CCGGAATGCCCTTTCTGAGC
## [5]    20 AGCTCCAGCCGTTTGACTTC
## [6]    20 GCGGAAAGTGAACTTAGATT
```

```
set.seed(10)
input_q <- random_qual(c(30,40), slength = 6, swidth = 20, 
           encod = "Sanger")

input_names <- seq_names(6)

# create ShortReadQ object
my_read <- ShortReadQ(sread = input, quality = input_q, id = input_names)

# apply the filter 
filtered3 <- fixed_filter(my_read, trim5 = 5)
sread(filtered3)
```

```
##   A DNAStringSet instance of length 6
##     width seq
## [1]    15 TGGTCCGGTGTTCTG
## [2]    15 ATAGGTACAGTCCAG
## [3]    15 GCCTCCCGCAGACGC
## [4]    15 CCGGAATGCCCTTTC
## [5]    15 AGCTCCAGCCGTTTG
## [6]    15 GCGGAAAGTGAACTT
```

```
filtered5 <- fixed_filter(my_read, trim3 = 5)
sread(filtered5)
```

```
##   A DNAStringSet instance of length 6
##     width seq
## [1]    15 CGGTGTTCTGGCGGA
## [2]    15 TACAGTCCAGTAATT
## [3]    15 CCGCAGACGCTGGGT
## [4]    15 ATGCCCTTTCTGAGC
## [5]    15 CAGCCGTTTGACTTC
## [6]    15 AAGTGAACTTAGATT
```

```
filtered3and5 <- fixed_filter(my_read, trim3 = 10, trim5 = 5)
sread(filtered3and5)
```

```
##   A DNAStringSet instance of length 6
##     width seq
## [1]     5 TTCTG
## [2]     5 TCCAG
## [3]     5 GACGC
## [4]     5 CTTTC
## [5]     5 GTTTG
## [6]     5 AACTT
```

Documentation of the function

- **length\_filter**

Removes sequences with a length lower than
minimum threshold value or/and higher than a maximum threshold value.

```
# create  ShortReadQ object width widths between 1 and 60
set.seed(10)
input <- random_length(10, widths = 1:60)
sread(input)
```

```
##   A DNAStringSet instance of length 10
##      width seq
##  [1]    31 TTCTGGCGGAATAGGTACAGTCCAGTAATTG
##  [2]    19 CCTCCCGCAGACGCTGGGT
##  [3]    26 CCGGAATGCCCTTTCTGAGCAGCTCC
##  [4]    42 AGCCGTTTGACTTCGCGGAAAGTGAACTTAGATTCGGTCCTG
##  [5]     6 AAACAC
##  [6]    14 GGTACTTCCACAGT
##  [7]    17 CAACCCGCCGACTTGGA
##  [8]    17 GAATTTATTAGCCGGGC
##  [9]    37 GGTTATTCCCCTAGTGATCTTACTAAGATTTGCAATA
## [10]    26 CCTAAGCGAAGTGACAGATATGTTCG
```

```
# apply the filter, removing sequences with  5>length> 30
filtered <- length_filter(input, rm.min = 5, rm.max = 30)
sread(filtered)
```

```
##   A DNAStringSet instance of length 7
##     width seq
## [1]    19 CCTCCCGCAGACGCTGGGT
## [2]    26 CCGGAATGCCCTTTCTGAGCAGCTCC
## [3]     6 AAACAC
## [4]    14 GGTACTTCCACAGT
## [5]    17 CAACCCGCCGACTTGGA
## [6]    17 GAATTTATTAGCCGGGC
## [7]    26 CCTAAGCGAAGTGACAGATATGTTCG
```

Documentation of the function

- **n\_filter**

Wrapper of the *ShortRead* nFilter function. Removes
all those sequences with a number of N’s > a given threshold.

```
# create 10 sequences of width 20
set.seed(10)
input <- random_seq(10, 20)
input
```

```
##   A DNAStringSet instance of length 10
##      width seq
##  [1]    20 TGGTCCGGTGTTCTGGCGGA
##  [2]    20 ATAGGTACAGTCCAGTAATT
##  [3]    20 GCCTCCCGCAGACGCTGGGT
##  [4]    20 CCGGAATGCCCTTTCTGAGC
##  [5]    20 AGCTCCAGCCGTTTGACTTC
##  [6]    20 GCGGAAAGTGAACTTAGATT
##  [7]    20 CGGTCCTGAAACACGGTACT
##  [8]    20 TCCACAGTCAACCCGCCGAC
##  [9]    20 TTGGAGAATTTATTAGCCGG
## [10]    20 GCGGTTATTCCCCTAGTGAT
```

```
# inject N's
set.seed(10)
input <- inject_letter_random(input, how_many_seqs = 1:5,
            how_many = 1:10)
input
```

```
##   A DNAStringSet instance of length 10
##      width seq
##  [1]    20 TGGTCCGGTGTTCTGGCGGA
##  [2]    20 ATAGGTACAGTCCAGTAATT
##  [3]    20 GCCTCCCGCAGACGCTGGGT
##  [4]    20 CCGGNATGCCCTTTCTGAGC
##  [5]    20 AGCTCCAGCCGTTTGACTTC
##  [6]    20 NCNNAANGTGNNCTTANATT
##  [7]    20 CGGTCCTGAAACACGGTACT
##  [8]    20 TCCACAGTCAACCCGCCGAC
##  [9]    20 TTGGAGAATTTATTAGCCGG
## [10]    20 GCGGTNANTCCNCTAGTGAT
```

```
#'  
hist(letterFrequency(input, "N"), breaks = 0:10, 
    main  = "Ns Frequency", xlab = "# Ns",
    col = "#1a81c2")
```

**Figure 15**: N’s histogram for the sequences before the filtering operation

```
# Create qualities, names and ShortReadQ object
set.seed(10)
input_q <- random_qual(10, 20)
input_names <- seq_names(10)
my_read <- ShortReadQ(sread = input, quality = input_q, id = input_names)

# Apply the filter 
filtered <- n_filter(my_read, rm.N = 3)
sread(filtered)
```

```
##   A DNAStringSet instance of length 9
##     width seq
## [1]    20 TGGTCCGGTGTTCTGGCGGA
## [2]    20 ATAGGTACAGTCCAGTAATT
## [3]    20 GCCTCCCGCAGACGCTGGGT
## [4]    20 CCGGNATGCCCTTTCTGAGC
## [5]    20 AGCTCCAGCCGTTTGACTTC
## [6]    20 CGGTCCTGAAACACGGTACT
## [7]    20 TCCACAGTCAACCCGCCGAC
## [8]    20 TTGGAGAATTTATTAGCCGG
## [9]    20 GCGGTNANTCCNCTAGTGAT
```

```
hist(letterFrequency(sread(filtered), "N"), 
    main = "Ns distribution", xlab = "",
    col = "#1a81c2")
```

**Figure 16**: N’s histogram for the sequences after the filtering operation

Documentation of the function

- **qmean\_filter**

Removes those sequences with quality < a give threshold.

```
# create 30 sequences of width 20, 15 with low quality and 15 with high quality
set.seed(10)
input <- random_seq(30, 20)

set.seed(10)
my_qual_H <- random_qual(c(30,40), slength = 15, swidth = 20,
                         encod = "Sanger")

set.seed(10)
my_qual_L <-   random_qual(c(5,30), slength = 15, swidth = 20, 
                           encod = "Sanger")
input_q<- c(my_qual_H, my_qual_L)

input_names <- seq_names(30)
my_read <- ShortReadQ(sread = input, quality = input_q, id = input_names)

# Plot of average qualities
qual_plot <- function(x, cutoff) {
q <- alphabetScore(x) / width(x)
plot(q, type="l", xlab = "Sequence", ylab= "Average quality", ylim = c(0, 40))
points(q, col = "#1a81c2", pch = 16, cex = 2)
lines(seq_along(q), rep(cutoff, length(q)), type="l", col = "red", lty=2)
text(length(q), cutoff+2, cutoff)
}

#' Average qualities before
qual_plot(my_read, cutoff = 30)
```

**Figure 17**: Average qualities before the filtering operation

```
# Apply the filter
filtered <- qmean_filter(my_read, minq = 30)

# Average qualities after
qual_plot(filtered, cutoff = 30)
```

**Figure 18**: Average qualities after the filtering operation

Documentation of the function

- **seq\_filter**

Removes sequences that match those passed as argument.

```
# Generate random sequences
set.seed(10)
input <- random_length(30, 3:7)

# Remove sequences that contain the following patterns:
rm.seq  = c("TGGTC", "CGGT", "GTTCT", "ATA")
match_before <- unlist(lapply(rm.seq, function(x) grep(x, 
as.character(sread(input)))))
match_before
```

```
## integer(0)
```

```
filtered <- seq_filter(input,rm.seq =  rm.seq)

# Verify that matching sequences were removed
match_after <- unlist(lapply(rm.seq, function(x) {
                grep(x, as.character(sread(filtered)))}))
match_after
```

```
## integer(0)
```

Documentation of the function

- **trim3q\_filter**

Removes from the 3’ ends in-tandem nucleotides with a quality
< a threshold value.

```
# Create 6 sequences of width 20
set.seed(10)
input <- random_seq(6, 20)
input
```

```
##   A DNAStringSet instance of length 6
##     width seq
## [1]    20 TGGTCCGGTGTTCTGGCGGA
## [2]    20 ATAGGTACAGTCCAGTAATT
## [3]    20 GCCTCCCGCAGACGCTGGGT
## [4]    20 CCGGAATGCCCTTTCTGAGC
## [5]    20 AGCTCCAGCCGTTTGACTTC
## [6]    20 GCGGAAAGTGAACTTAGATT
```

```
# Create Phred+33 qualities of width 15 and paste to qualities of length 
# 5 used for the tails.
# for three of the sequences, put low qualities in tails
set.seed(10)
my_qual <- random_qual(c(30,40), slength = 6, swidth = 15, 
                        encod = "Sanger")
set.seed(10)
tails <-   random_qual(c(30,40), slength = 6, swidth = 5, 
                        encod = "Sanger")

# Low quality tails in sequences 2, 3 & 4
set.seed(10)
tails[2:4] <- random_qual(c(3, 20), slength = 3, swidth = 5,
                        encod = "Sanger")
my_qual <- paste0(my_qual, tails)
input_q <- BStringSet(my_qual)
input_q
```

```
##   A BStringSet instance of length 6
##     width seq
## [1]    20 EGFEDIBEH@C@DD?EAAID
## [2]    20 I?EGDHIBEG?BHFG,%),4
## [3]    20 ACCFBBFCI?I@HBC402+,
## [4]    20 CGIAFGB@?AIDF@I14)2+
## [5]    20 IB@ACAAC?AGEDDHC?BEB
## [6]    20 BH?GFFHHG?DABECFEEDE
```

```
# Watch qualities before filtering
as.matrix(PhredQuality(input_q))
```

```
##      [,1] [,2] [,3] [,4] [,5] [,6] [,7] [,8] [,9] [,10] [,11] [,12] [,13]
## [1,]   36   38   37   36   35   40   33   36   39    31    34    31    35
## [2,]   40   30   36   38   35   39   40   33   36    38    30    33    39
## [3,]   32   34   34   37   33   33   37   34   40    30    40    31    39
## [4,]   34   38   40   32   37   38   33   31   30    32    40    35    37
## [5,]   40   33   31   32   34   32   32   34   30    32    38    36    35
## [6,]   33   39   30   38   37   37   39   39   38    30    35    32    33
##      [,14] [,15] [,16] [,17] [,18] [,19] [,20]
## [1,]    35    30    36    32    32    40    35
## [2,]    37    38    11     4     8    11    19
## [3,]    33    34    19    15    17    10    11
## [4,]    31    40    16    19     8    17    10
## [5,]    35    39    34    30    33    36    33
## [6,]    36    34    37    36    36    35    36
```

```
# Create names and ShortReadQ object
input_names <- seq_names(6)
my_read <- ShortReadQ(sread = input, quality = input_q, id = input_names)

# Apply the filter 
filtered <- trim3q_filter(my_read, rm.3qual = 28)
sread(filtered)
```

```
##   A DNAStringSet instance of length 6
##     width seq
## [1]    20 TGGTCCGGTGTTCTGGCGGA
## [2]    15 ATAGGTACAGTCCAG
## [3]    15 GCCTCCCGCAGACGC
## [4]    15 CCGGAATGCCCTTTC
## [5]    20 AGCTCCAGCCGTTTGACTTC
## [6]    20 GCGGAAAGTGAACTTAGATT
```

Documentation of the function

- **unique\_filter**

Wrapper of the *ShortRead* occurrenceFilter function.
that removes duplicated sequences.

```
# Create duplicated sequences
s <- random_seq(10, 10)
s <- sample(s, 30, replace = TRUE)

# Create a ShortReadQ object
q <- random_qual(30, 10)
n <- seq_names(30)
my_read <- ShortReadQ(sread = s, quality = q, id = n)

# Check presence of duplicates
isUnique(as.character(sread(my_read)))
```

```
##  [1] FALSE FALSE FALSE FALSE FALSE FALSE FALSE FALSE FALSE  TRUE FALSE FALSE
## [13] FALSE FALSE FALSE FALSE  TRUE FALSE FALSE FALSE FALSE FALSE FALSE FALSE
## [25] FALSE FALSE FALSE FALSE FALSE FALSE
```

```
# Apply the filter
filtered <- unique_filter(my_read)
isUnique(as.character(sread(filtered)))
```

```
##  [1] TRUE TRUE TRUE TRUE TRUE TRUE TRUE TRUE TRUE TRUE
```

Documentation of the function

## 4.2 Auxiliary functions

- **random\_seq**

Create a vector of random sequences, for a set of specificied parameters.

Documentation of the function

- **random\_qual**

Create a vector of random qualities for a given encoding and a set
of specified parameters.

Documentation of the function

- **seq\_names**

Create a vector of names for a set of sequences.

Documentation of the function

- **random\_length**

Create a set of sequences with random lengths.

Documentation of the function

- **inject\_letter\_random**

Inject a character (e.g., ‘N’) at random positions, given a set of parameters.

Documentation of the function

- **check\_encoding**

The function allows to check quality encoding. It detects encodings with
the following formats:

| Format | Expected range |
| --- | --- |
| Sanger | [0, 40] |
| Illumina 1.8 | [0, 41] |
| Illumina 1.5 | [0, 40] |
| Illumina 1.3 | [3, 40] |
| Solexa | [-5, 40] |

Documentation of the function

# 5 Contact information

Mantainer: Leandro Roser - learoser@gmail.com
