# Supplementary figures and images for: FastqCleaner: an interactive Bioconductor application for quality-control, filtering and trimming of FASTQ files
Source: BMC Bioinformatics. 2019 Jun 28;20:361. doi: 10.1186/s12859-019-2961-8 (PMC6599294; doi:10.1186/s12859-019-2961-8)

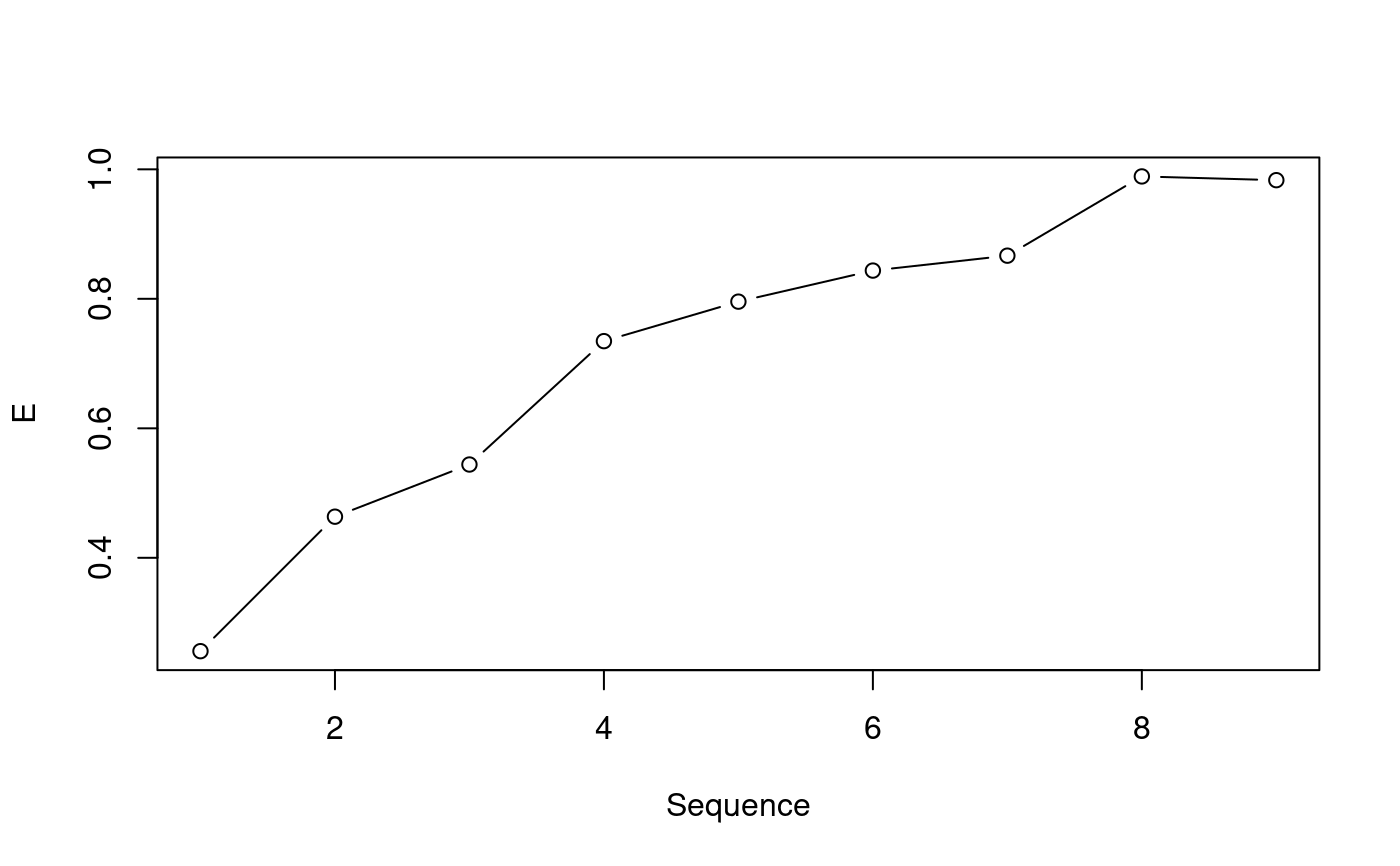

Supplement: Supplementary file 3 — Source code of FastqCleaner. (GZ 3273 kb) [file 12859_2019_2961_MOESM3_ESM.gz › FastqCleaner/inst/application/www/help/docs/reference/complex_filter-1.png]

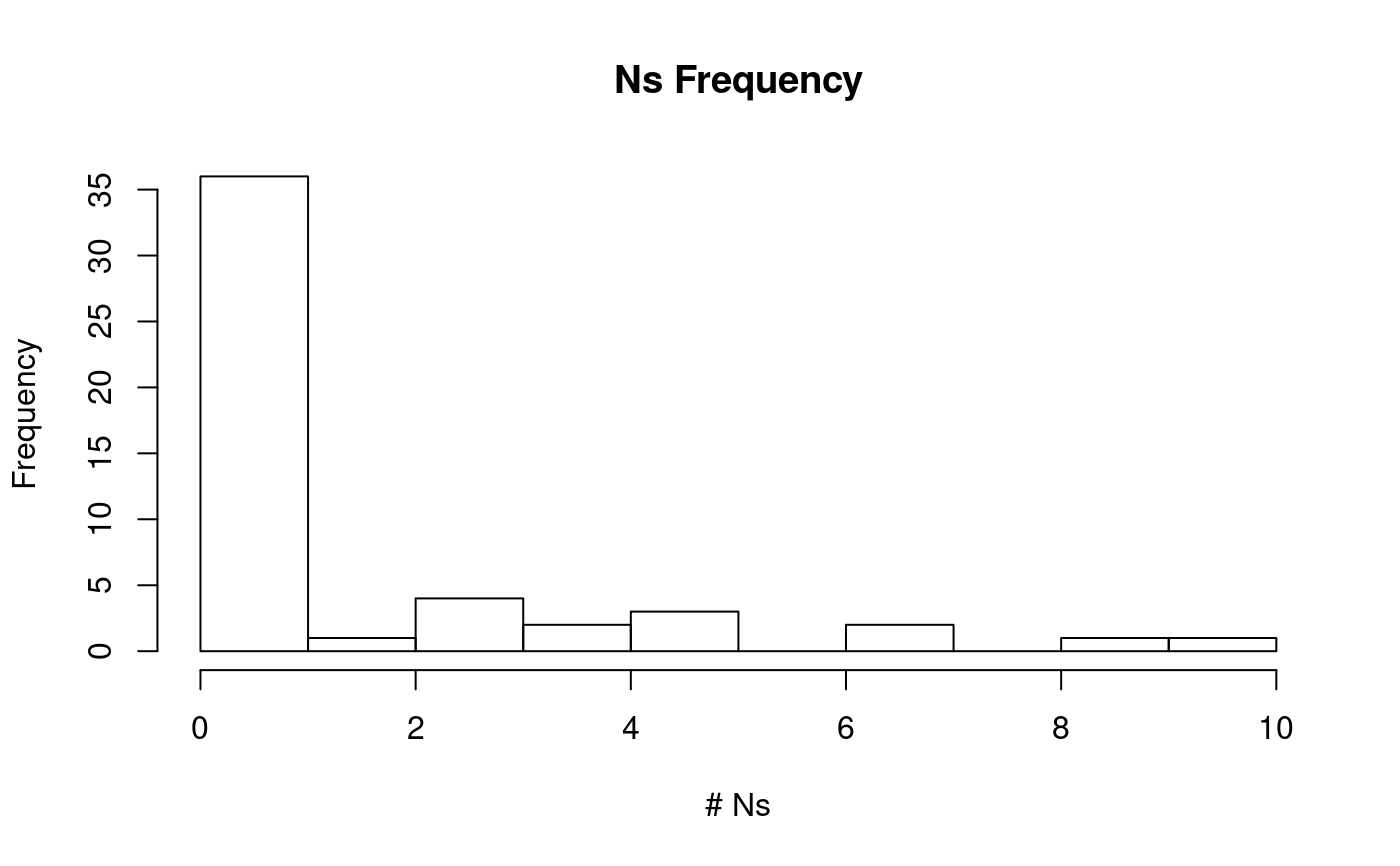

Supplement: Supplementary file 3 — Source code of FastqCleaner. (GZ 3273 kb) [file 12859_2019_2961_MOESM3_ESM.gz › FastqCleaner/inst/application/www/help/docs/reference/n_filter-1.png]

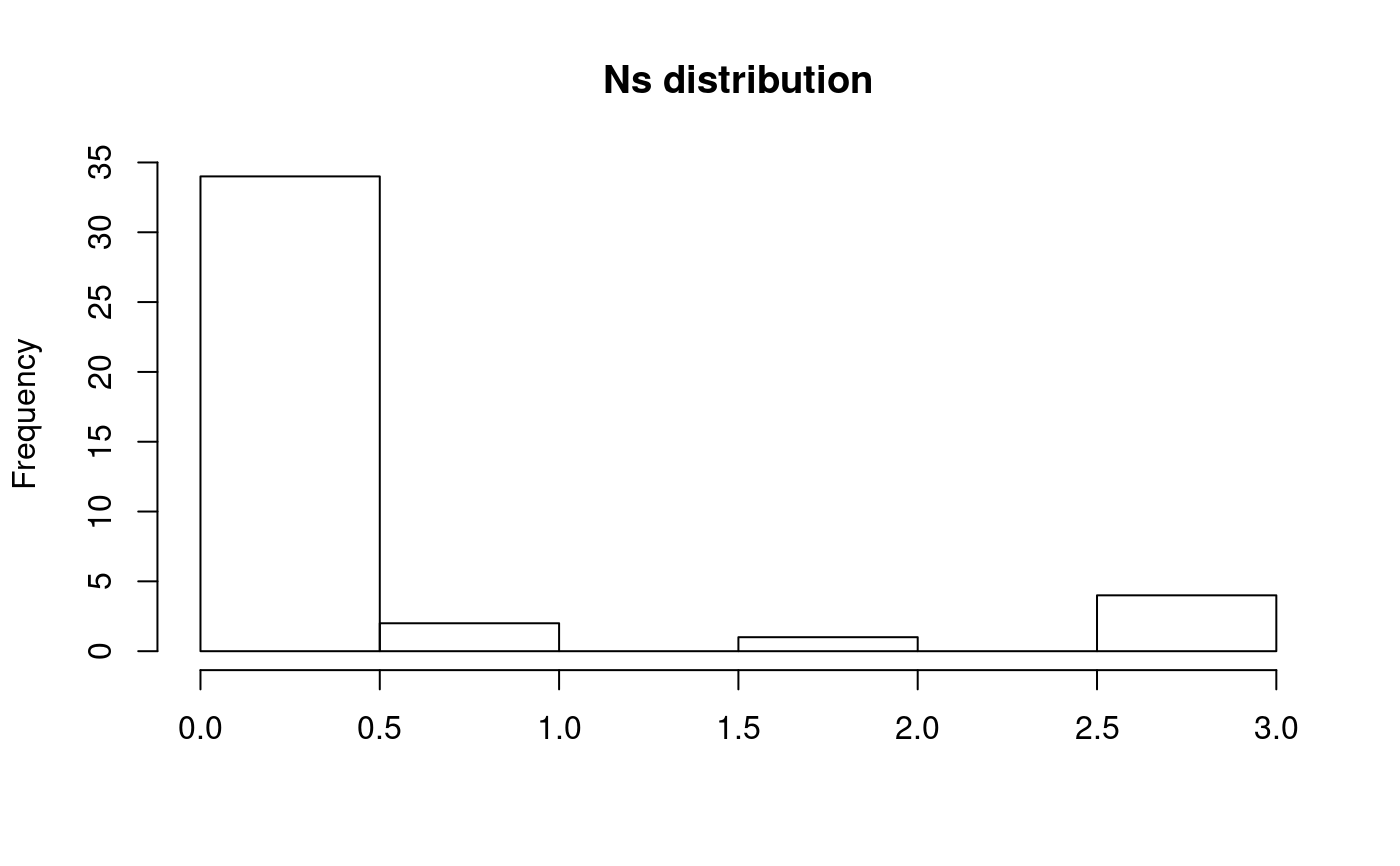

Supplement: Supplementary file 3 — Source code of FastqCleaner. (GZ 3273 kb) [file 12859_2019_2961_MOESM3_ESM.gz › FastqCleaner/inst/application/www/help/docs/reference/n_filter-2.png]

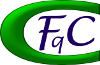

Supplement: Supplementary file 3 — Source code of FastqCleaner. (GZ 3273 kb) [file 12859_2019_2961_MOESM3_ESM.gz › FastqCleaner/inst/application/www/images/icon.png]

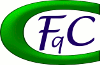

Supplement: Supplementary file 3 — Source code of FastqCleaner. (GZ 3273 kb) [file 12859_2019_2961_MOESM3_ESM.gz › FastqCleaner/inst/application/www/images/icon2.gif]

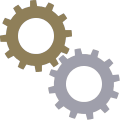

Supplement: Supplementary file 3 — Source code of FastqCleaner. (GZ 3273 kb) [file 12859_2019_2961_MOESM3_ESM.gz › FastqCleaner/inst/application/www/images/loading.gif]

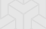

Supplement: Supplementary file 3 — Source code of FastqCleaner. (GZ 3273 kb) [file 12859_2019_2961_MOESM3_ESM.gz › FastqCleaner/inst/application/www/images/pattern.png]

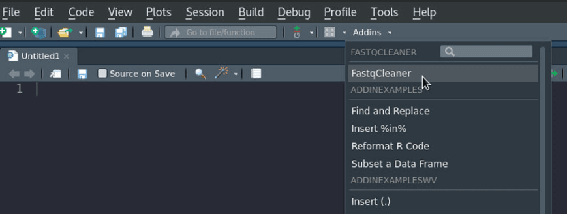

Supplement: Supplementary file 3 — Source code of FastqCleaner. (GZ 3273 kb) [file 12859_2019_2961_MOESM3_ESM.gz › FastqCleaner/vignettes/images/Fig1.png]

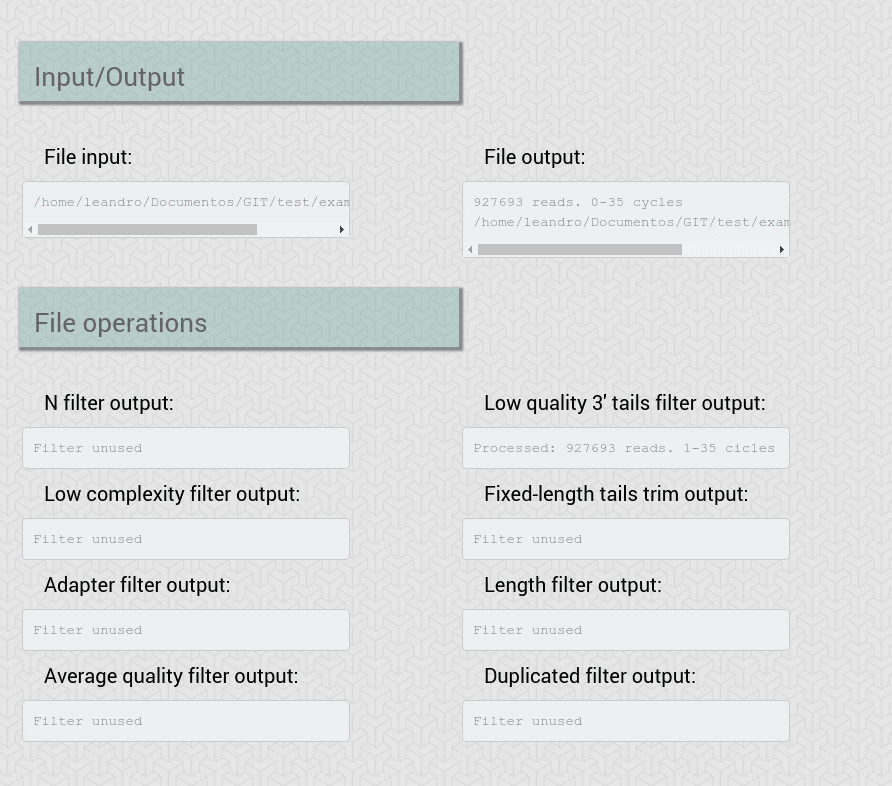

Supplement: Supplementary file 3 — Source code of FastqCleaner. (GZ 3273 kb) [file 12859_2019_2961_MOESM3_ESM.gz › FastqCleaner/vignettes/images/Fig10.png]

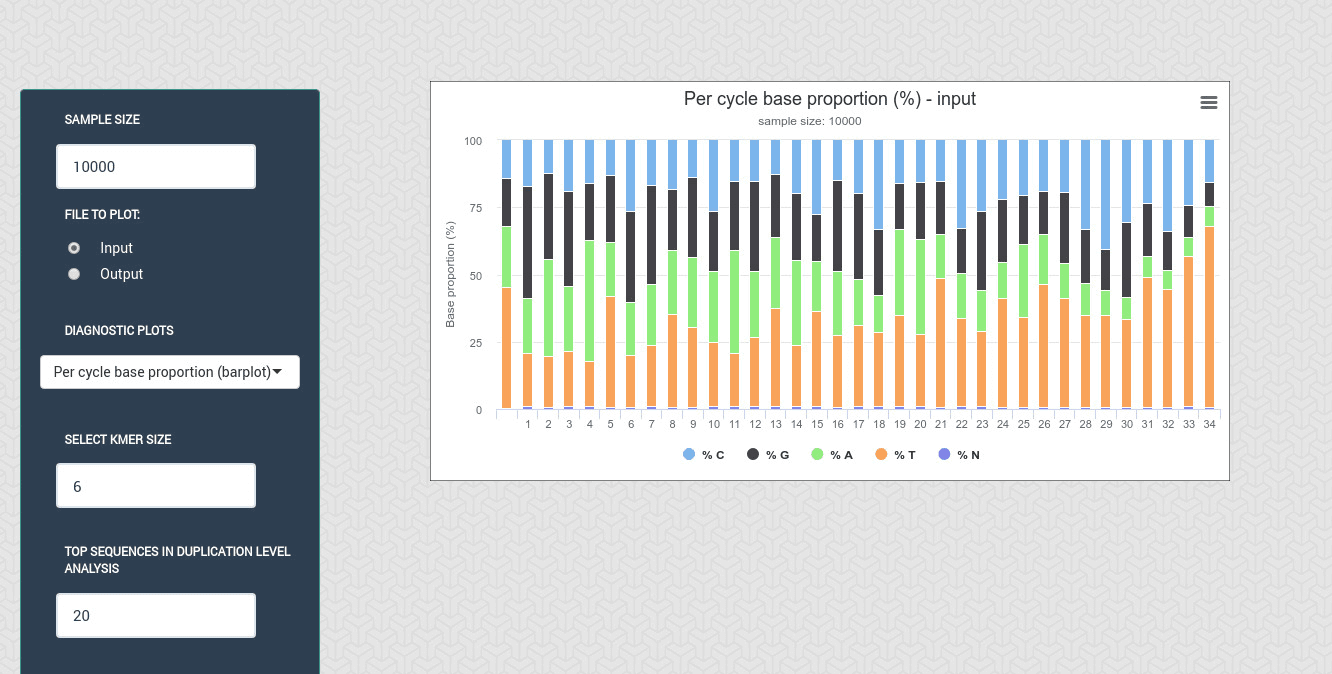

Supplement: Supplementary file 3 — Source code of FastqCleaner. (GZ 3273 kb) [file 12859_2019_2961_MOESM3_ESM.gz › FastqCleaner/vignettes/images/Fig11.png]

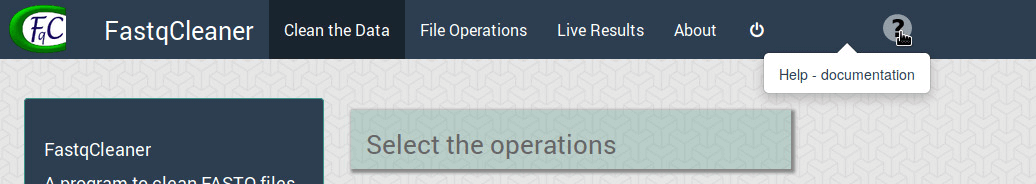

Supplement: Supplementary file 3 — Source code of FastqCleaner. (GZ 3273 kb) [file 12859_2019_2961_MOESM3_ESM.gz › FastqCleaner/vignettes/images/Fig12.png]

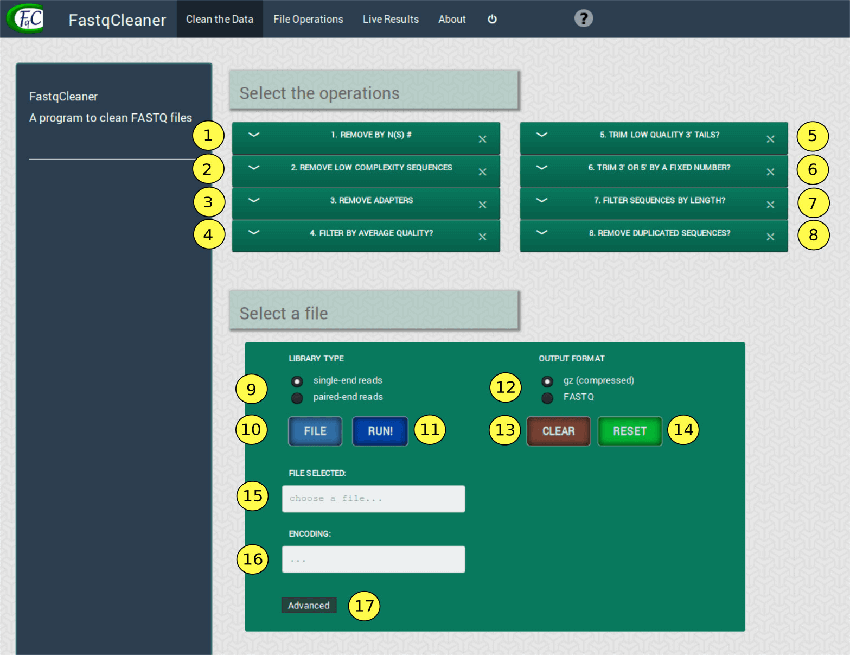

Supplement: Supplementary file 3 — Source code of FastqCleaner. (GZ 3273 kb) [file 12859_2019_2961_MOESM3_ESM.gz › FastqCleaner/vignettes/images/Fig2.png]

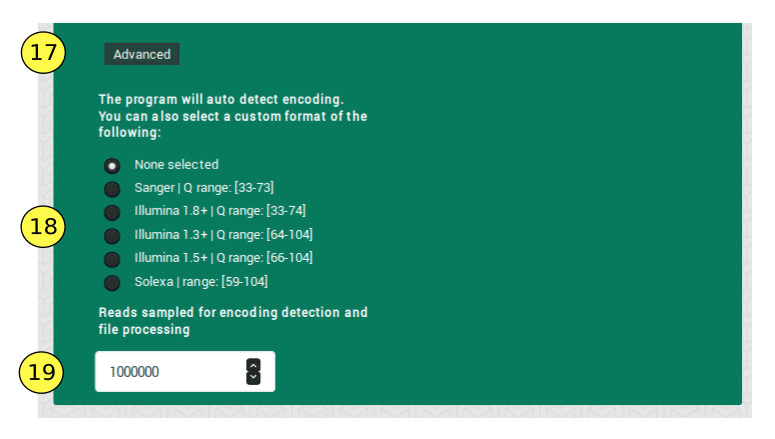

Supplement: Supplementary file 3 — Source code of FastqCleaner. (GZ 3273 kb) [file 12859_2019_2961_MOESM3_ESM.gz › FastqCleaner/vignettes/images/Fig3.png]

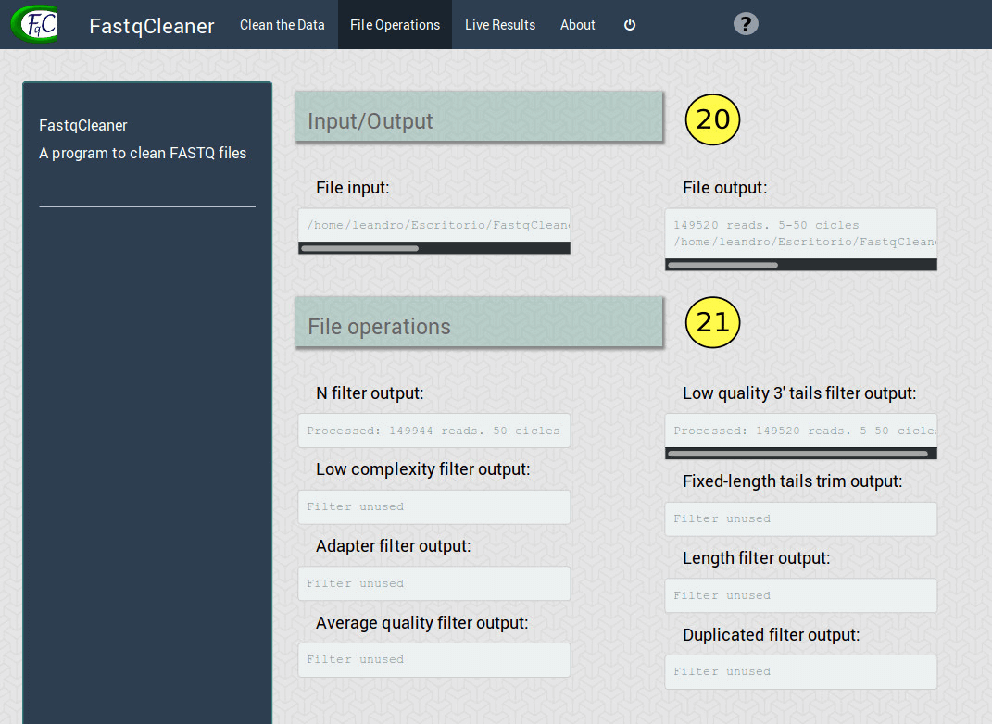

Supplement: Supplementary file 3 — Source code of FastqCleaner. (GZ 3273 kb) [file 12859_2019_2961_MOESM3_ESM.gz › FastqCleaner/vignettes/images/Fig4.png]

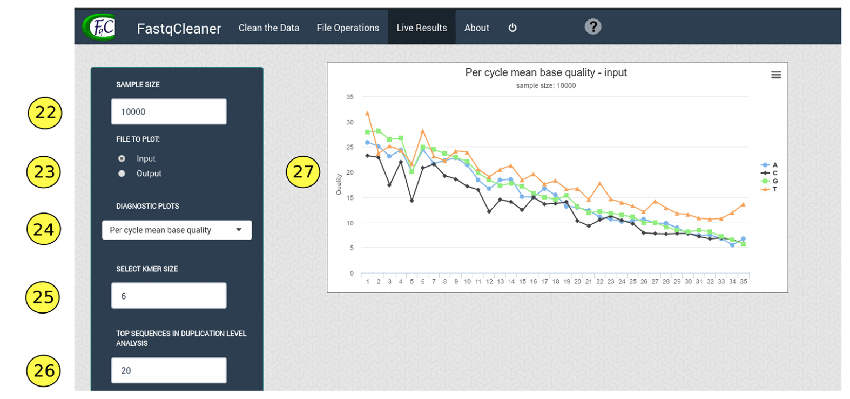

Supplement: Supplementary file 3 — Source code of FastqCleaner. (GZ 3273 kb) [file 12859_2019_2961_MOESM3_ESM.gz › FastqCleaner/vignettes/images/Fig5.png]

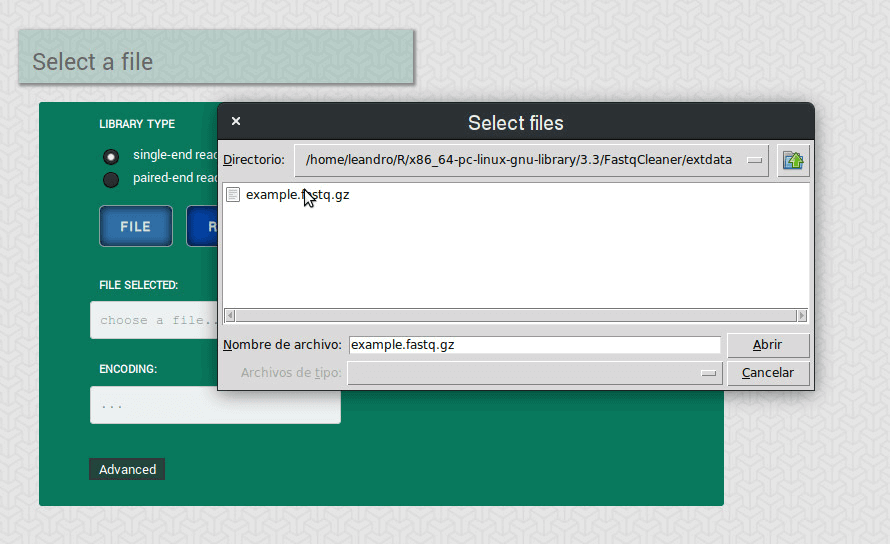

Supplement: Supplementary file 3 — Source code of FastqCleaner. (GZ 3273 kb) [file 12859_2019_2961_MOESM3_ESM.gz › FastqCleaner/vignettes/images/Fig6.png]

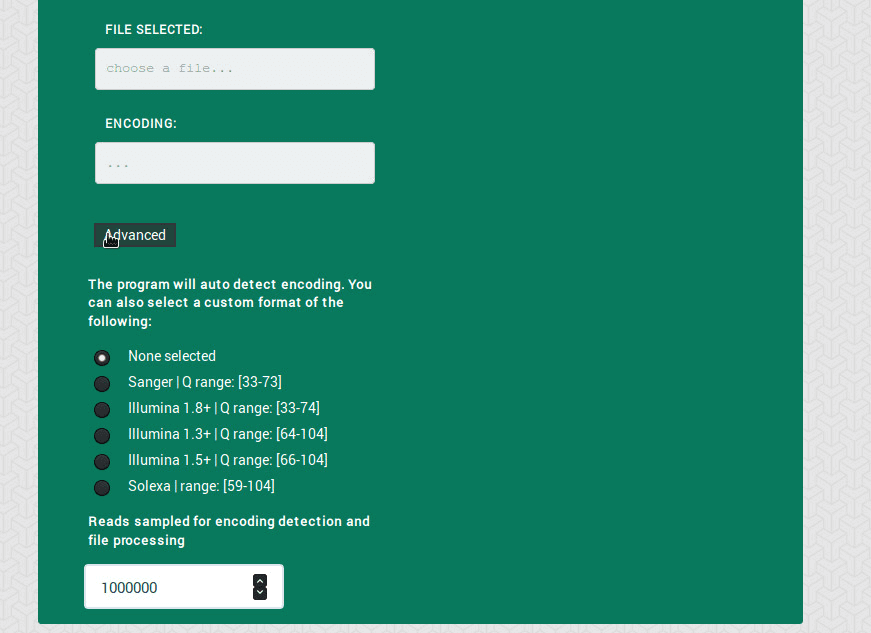

Supplement: Supplementary file 3 — Source code of FastqCleaner. (GZ 3273 kb) [file 12859_2019_2961_MOESM3_ESM.gz › FastqCleaner/vignettes/images/Fig7.png]

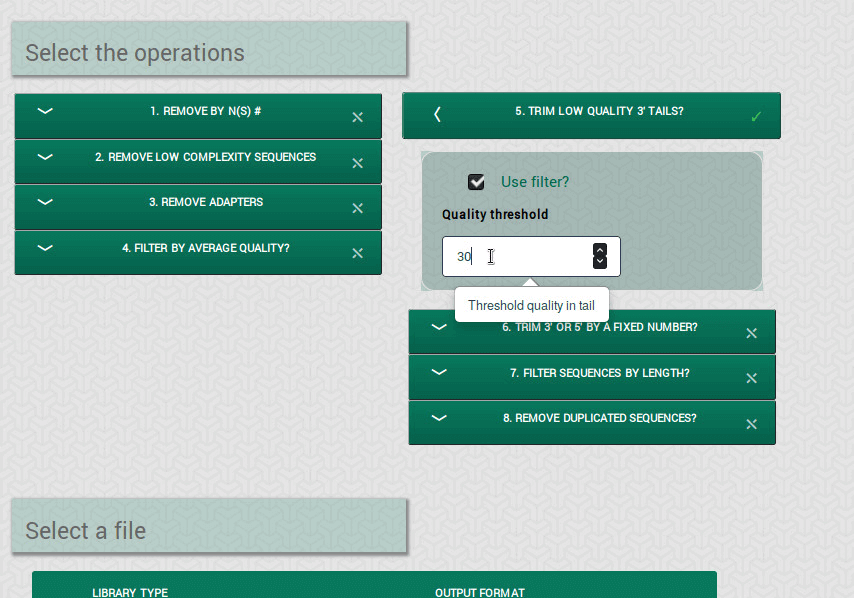

Supplement: Supplementary file 3 — Source code of FastqCleaner. (GZ 3273 kb) [file 12859_2019_2961_MOESM3_ESM.gz › FastqCleaner/vignettes/images/Fig8.png]

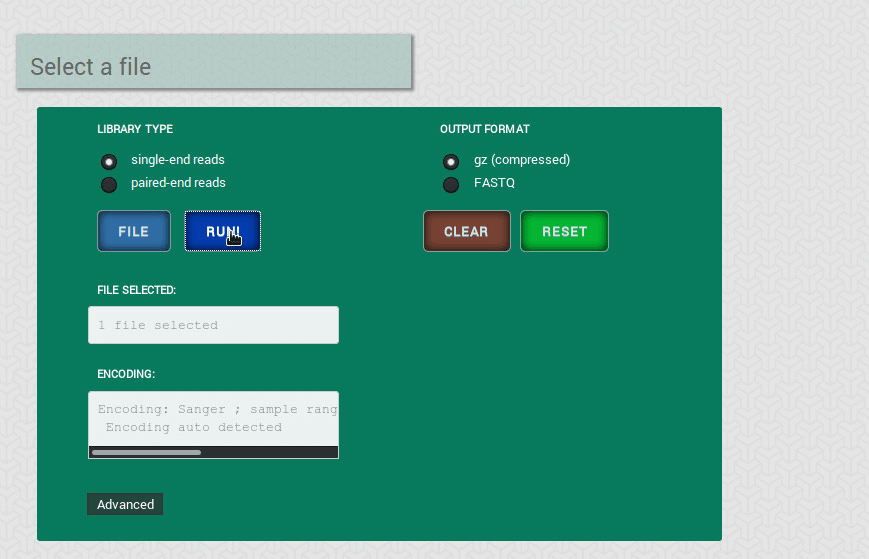

Supplement: Supplementary file 3 — Source code of FastqCleaner. (GZ 3273 kb) [file 12859_2019_2961_MOESM3_ESM.gz › FastqCleaner/vignettes/images/Fig9.png]
